# PI3Kα inhibitor CYH33 triggers antitumor immunity in murine breast cancer by activating CD8^+^T cells and promoting fatty acid metabolism

**DOI:** 10.1136/jitc-2021-003093

**Published:** 2021-08-09

**Authors:** Pu Sun, Xi Zhang, Rong-Jing Wang, Qing-Yang Ma, Lan Xu, Yi Wang, Hui-Ping Liao, Hai-Long Wang, Lan-Dian Hu, Xiangyin Kong, Jian Ding, Ling-Hua Meng

**Affiliations:** 1 Division of Anti-tumor Pharmacology, Shanghai Institute of Materia Medica, Chinese Academy of Sciences, Shanghai, China; 2 University of Chinese Academy of Sciences, Beijing, China; 3 Key Laboratory of Tissue Microenvironment and Tumor, Shanghai Institute of Nutrition and Health, Chinese Academy of Sciences, Shanghai, China; 4 Division of Anti-tumor Pharmacology, State Key Laboratory of Drug Research, Shanghai Institute of Materia Medica, Chinese Academy of Sciences, Shanghai, China

**Keywords:** tumor microenvironment, immunomodulation, immunologic memory, lymphocytes, tumor-infiltrating, macrophages

## Abstract

**Background:**

The phosphatidylinositol 3-kinase (PI3K) is frequently hyperactivated in cancer and plays important roles in both malignant and immune cells. The effect of PI3Kα inhibitors on the tumor microenvironment (TME) remains largely unknown. Here, we investigated the modulation of the TME by a clinical PI3Kα-specific inhibitor CYH33.

**Methods:**

The activity of CYH33 against a panel of murine tumors in the immune-competent context or athymic mice was detected. Single-cell RNA sequencing and multi-parameter flow cytometry were performed to determine the immune profiling of TME. The effect of CYH33 on immune cells was conducted with primary murine cells.

**Results:**

CYH33 exhibited more potent antitumor activity in immune-competent context. CYH33 enhanced the infiltration and activation of CD8^+^T and CD4^+^T cells, while attenuating M2-like macrophages and regulatory CD4^+^T cells. Increase in memory T cells was confirmed by the induction of long-term immune memory on CYH33 treatment. Mechanistically, CYH33 relieved the suppressed expansion of CD8^+^T cells via preferential polarization of the macrophages to the M1 phenotype. CYH33 promoted fatty acid (FA) metabolism in the TME, while FA enhanced the activity of CD8^+^T cells in vitro. The combination of CYH33 with the FA synthase (FASN) inhibitor C75 synergistically inhibited tumor growth with enhanced host immunity.

**Conclusions:**

CYH33 induces immune activation and synergizes with FASN inhibitor to further promote the antitumor immunity, which gains novel insights into how PI3K inhibitors exert their activity by modulating TME and provides a rationale for the concurrent targeting of PI3K and FASN in breast cancer treatment.

## Introduction

The phosphatidylinositol 3-kinase (PI3K)/protein kinase B (AKT)/mammalian target of rapamycin pathway is one of the most frequently dysregulated signaling pathways in human cancer.^1^ PI3Ks are lipid kinases that phosphorylate the 3’-hydroxyl group of the inositol ring of phosphatidylinositol and regulate cell growth, proliferation, survival, motility, and metabolism.^2 3^ Based on substrate preference and sequence homology, PI3Ks can be divided into three classes. Class I PI3Ks are further divided into PI3Kα, PI3Kβ, PI3Kδ, and PI3Kγ.^4^ As PI3K is validated as a promising target for cancer therapy, significant progress has been made in the development of novel PI3K inhibitors. To date, four PI3K inhibitors have been approved for cancer treatment. The PI3Kδ inhibitor idelalisib was the first one approved for the treatment of chronic lymphocytic leukemia in combination with rituximab in 2014,^5 6^ followed by copanlisib and duvelisib, approved in 2017 and 2018, respectively.^7 8^ The PI3Kα inhibitor alpelisib was the first PI3K inhibitor approved for the treatment of solid tumor, which combines endocrine therapy for treatment of breast cancer.^9^ A number of PI3K inhibitors are being evaluated in clinical trials as monotherapy or combinatorial regimens.

Tumor progression depends not only on the intrinsic hallmarks of cancer cells but also on the formation of a milieu that escapes immune surveillance. Regulatory immune cells including myeloid-derived suppressor cells (MDSCs), M2-like tumor-associated macrophages (TAMs), and CD4^+^CD25^high^FoxP3^+^ regulatory T cells (Tregs) contribute to the inhibition of cytotoxic CD8^+^T cells and tumor progression.^10 11^ Although the effect of PI3K inhibitors on cancer cells has been extensively studied, their effect on cancer-associated immunity remains largely unknown. Emerging evidence has highlighted the potential novel mechanisms underlying the antitumor effect of PI3K inhibitors.^12^ For example, PI3Kγ inhibition delay tumor growth by inhibiting the function of macrophages and activating CD8^+^T cells.^13–15^ PI3Kδ inhibitors inhibit the proliferation of Tregs and attenuate their immunosuppressive effect.^16 17^ PI3Kα is the only isoform that frequently mutated and abnormally activated in solid tumors.^18^ The intrinsic PI3Kα activation in malignant cells may mediate the suppressive tumor microenvironment (TME). For example, the activation of PI3Kα limited the infiltration and function of T cells, and contributed to the suppressed expression of major histocompatibility complex I and cluster of differentiation 80 in tumor cells.^19^ Despite the potential role of PI3Kα in the TME, the efficacy and mechanism of action of PI3Kα inhibitors are typically studied in athymic nude mice in preclinical settings. Elucidating the action of PI3Kα inhibitors in the immune-competent context will facilitate a better understanding of the impact of the PI3K pathway on the crosstalk between the TME and cancer cells, and allow further exploration of mechanism-driven drug combinations to potentiate the efficacy.

CYH33 is a novel PI3Kα-selective inhibitor discovered by our group with a distinctive structure, which exhibits superior anticancer activity both in vitro and in vivo compared with alpelisib.^20–23^ A phase I clinical study of CYH33 and a phase Ib study of CYH33 in combination with olaparib in patients with advanced solid tumors (NCT03544905, NCT04586335) are ongoing. Our previous studies demonstrated that CYH33 significantly inhibited the proliferation of a panel of human breast cancer cells and non-small cell lung cancer cells by the induction of G_1_ phase arrest.^20 24^ Notably, we found that CYH33 enhanced the activity of radiation against esophageal squamous cell carcinoma by abrogating the radiation-induced phosphorylation of AKT and infiltration of M2-like macrophages,^21^ indicating its potential effect on the TME.

In this study, we screened the activity of CYH33 in a panel of murine tumor cells and allografts inoculated in immune-competent mice. Deep immune profiling of tumor tissue by single-cell RNA-seq (scRNA-seq) and flow cytometry demonstrated that CYH33 enhanced the infiltration and activation of T cells as well as modulated the myeloid cells. CYH33 may release the suppression of CD8^+^T cells mediated by M2-like macrophages by reprogramming macrophages. CYH33 treatment also upregulated fatty acid (FA) metabolism, which was accompanied with increased free FA (FFA) level and the activity of CD8^+^T cells. The combination of CYH33 with the FA synthase (FASN) inhibitor C75 further enhanced the immunogenic effect of CYH33 and led to synergistic antitumor efficacy.

## Materials and methods

### Compounds

CYH33 was provided by Shanghai HaiHe Biopharma Co., Ltd. Alpelisib was purchased from Dalian Meilun Biotechnology (Dalian, China, No. MB5532). C75 was purchased from MedChemExpress (New Jersey, USA, No. HY-12364). All compounds were dissolved in dimethyl sulfoxide (DMSO, Sigma, St. Louis, Missouri, USA) at the concentration of 10 mM and stored at −20°C. For in vivo experiments, CYH33 was dissolved in normal saline containing 0.5% Tween 80 (v/v; Sangon Biotech, Shanghai, China) and 1% CMC-Na (m/v; SINOPHARM, Beijing, China).

### Cell lines and culture

4T1, EMT6, LLC1, B16F10, KLN-205, A20, and CT26 cells were obtained from the American Type Culture Collection (Manassas, Virginia, USA). Colon 26 cells were obtained from the Japanese Collection of Research Bioresources Cell Bank (JCRB, Osaka, Japan). PY8119 cells were generously provided by Dr. Suling Liu (Shanghai Cancer Center & Institutes of Biomedical Sciences, Fudan University). U14 and MC38 cells were obtained from Cobioer Biosciences (Shanghai, China). All cells were maintained in the medium suggested by the suppliers.

### Cell proliferation assay

Cell proliferation was evaluated by sulforhodamine B (SRB) assay as described previously^25^ or Cell Counting Kit-8 (CCK-8, Dojindo Laboratories, Kumamoto, Japan) according to the manufacture’s instruction. The inhibitory rate was calculated using the formula: (OD_control cells_-OD_treated cells_)/(OD_control cells_-OD_Day0 cells_)×100%. GI_50_ values were obtained using four parameter concentration-response curve fitting with SoftMaxPro (Molecular Devices, California, USA).

### Western blotting

Cell lysates were prepared and subjected to western blotting with primary antibodies against AKT (No. 4691), phosphor-AKT (Ser473, No.4060), phosphor-AKT (Thr308, No. 13038) (Cell Signaling Technology, Danvers, Massachusetts, USA), and β-Actin (ProteinTech, Chicago, Illinois, USA, No. 60 008–1). Images were captured with the ImageQuant LAS 4000 system (GE, Boston, USA).

### Animal studies

Female Balb/c, Balb/c nude, C57BL/6, and DBA/2 mice aged 6–8 weeks old were obtained from Shanghai Institute of Materia Medica (Shanghai, China). Tumor cells were subcutaneously inoculated into the right side of flank. Animals were randomized to receive vehicle or tested compounds when tumor volume reaches about 50 mm^3^. Mice were administered orally with vehicle, CYH33, and alpelisib or intraperitoneally injected with C75. Body weight and tumor volume were measured and recorded twice per week. The tumor volume (V) was calculated using the formula: V=0.5 × length (mm) ×width (mm)^2^. Relative tumor volume (RTV) was calculated as the ratio of tumor volume at the given time point versus tumor volume at the initial treatment. The treatment to control ratio (T/C) was calculated as the following formula: T/C (%) = (T_RTV_/C_RTV_)×100%, where T_RTV_ and C_RTV_ represented the RTV of treatment and vehicle control group, respectively. The synergy of the combination of CYH33 and C75 in vivo was determined using the Bliss independence model, namely fractional product method, as described previously.^24 26^

### Generation of single-cell suspension from tumor tissue

Tumor-bearing mice were sacrificed after drug treatment for 7 days. Single-cell suspension was prepared as previously described with modifications.^27^ Briefly, primary tumors were harvested, cut into small species and then suspended in tumor dissociation buffer. Tumor sample was homogenized and incubated for 30 min at 37 ℃. The dissociated cells were filtered through a 70 µM cell strainer. Erythrocytes were lysed with ACK lysis buffer (ThermoFisher, Waltham, Massachusetts, USA, No. A1049201) on ice for 3 min and cells were washed with PBS and used for further analysis.

### Immunostaining and FACS analysis

Single-cell suspension prepared from tumor tissue were pre-incubated with anti-CD16/32 monoclonal antibody (FcR-blocking, BD Biosciences, San Jose, California, USA, clone 2.4G2) and then stained with antibodies against membrane markers for 45 min at 4℃. Dead cells were marked using Fixable Viability Stain 510 (BD Pharmingen, San Diego, California, USA, No. 564406). For intracellular staining, cells were fixed and permeabilized using the Foxp3/Transcription Factor Staining Buffer Set (eBioscience, San Diego, California, USA, No. 00-5523-00) or the Intracellular Fixation & Permeabilization Buffer Set (eBioscience, No. 88-8824-00) following the manufacture’s instruction. Antibodies were added and incubated for 1 hour at 4℃. Intracellular cytokine staining was performed 4–6 hours after ex vivo stimulation with leucocyte activation cocktail in the presence of GolgiStop (BD Pharmingen, No. 550583) at 37℃. All the fluorescently labeled antibodies used for staining are listed in online supplemental table S1. Data were collected with a BD LSRFortessa and analyzed using FlowJo (V.10.0). According to the isotype and fluorescence-minus-one, the gating strategies for flow cytometry were showed in online supplemental figure S4.

10.1136/jitc-2021-003093.supp1 Supplementary data

10.1136/jitc-2021-003093.supp2 Supplementary data

### scRNA-seq and analysis

Tumor samples were processed using the mouse tumor dissociation kit (Miltenyi Biotec, Bergisch Gladbach, Germany, No. 130-096-730). CD45^+^ tumor-infiltrating leucocytes were enriched from the cell suspension using CD45 (TIL) MicroBeads (Miltenyi Biotec, No. 130-110-618) according to the manufacture’s instruction. The single-cell suspension of tumor cells or isolated CD45^+^ cells with high cell viability was loaded and single cells were captured by the BD Rhapsody Single-cell Analysis System. Single-cell cDNA was prepared using BD Rhapsody cDNA Kit (No. 633773). The constructed cDNA libraries were sequenced with the Illumina NovaSeq PE150 platform and each unique molecular identifier was captured at least six times. For analysis, the sequencing data were processed using Rhapsody pipeline (V.1.8). The expression matrix of each sample was used for further analysis using the R package Seurat (V.3.1.4)^28^ in R (V.3.5.1). As a quality-control step, low-quality cells (gene number <100, mitochondrial gene percentage >80%) and duplicated cells were excluded. Gene set variation analysis implemented in the GSVA package (V.4.0)^29^ was used for gene set enrichment analysis (GSEA). The cell cycle analysis was performed using Seurat package according to the gene lists of cell cycles integrated in Seurat.

### Depletion of T cell or macrophage in vivo

The depletion of T cells and macrophages were conducted as previously described.^30^ Mice-bearing 4T1 tumor were intraperitoneally injected twice before the initial treatment at a 72 hours interval with anti-CD8 antibodies (400 µg, BioXCell, West Lebanon, USA, clone 2.43), anti-CD4 antibodies (400 µg, BioXCell, clone GK1.5), or clodrosome (50 mg/kg, Standard Macrophage Depletion Kit, Encapsula NanoSciences, Brentwood, Tennessee, USA, No. CLD-8901), respectively. Equal amounts of Rat IgG2b isotype antibodies (BioXcell) or Encapsome (Encapsula NanoSciences) were injected as a control. To validate the depletion of target cells, splenocytes were collected from spleens 48 hours after the last injection and stained with antibodies against CD4, CD8a, or CD11b and F4/80. Cells were analyzed with flow cytometry. During the drug treatment, antibodies (200 µg) or liposome (25 mg/kg) were injected every 3 days as shown in online supplemental figure 5A.

### The proliferation of primary murine T cell

Fresh murine spleen tissue was cut into small species and subjected to digestion using mouse Spleen Dissociation Kit (Miltenyi Biotec, No. 130-095-926) according to the manufacture’s protocol. CD8^+^T, CD4^+^T, and CD4^+^CD25^−^T cells were enriched from cell suspension using EasySep Mouse CD8^+^T cell Isolation Kit (Stemcell Technology, Vancouver, Canada, No. 19 853), Mouse CD4^+^T cell Isolation Kit (Miltenyi Biotec, No. 130-104-453), or EasySep Mouse CD4^+^CD25^+^ Tregs Isolation Kit II (Stemcell Technology, No. 18 783), respectively. Tregs were induced from CD4^+^CD25^-^T cells for 5 days as described.^31^

For proliferation assay, T cells were labeled with 5 µM carboxyfluorescein succinimidyl amino ester (CFSE, BD Horizon, Franklin Lakes, New Jersey, USA, No. 565082) for 15 min in serum-free medium. CD8^+^T cells were cultured for 72 hours in 1640 medium supplemented with 10% fetal bovine serum (FBS) and 100 U/mL of interleukin 2 (IL-2) (Peprotech, Rocky Hill, NJ, USA, No. 212-12-50). CD4^+^T cells and Tregs were stimulated and cultured for 72 hours as described.^31 32^

To detect the effect of FFA on the CD8^+^T cells, palmitic acid (Sigma, No. P5585)/bovine serum albumin (BSA, Beyotime Biotechnology, Shanghai, China, No. ST025) complex was prepared as reported.^33^ The water-soluble oleic acid (Sigma, No. O1257) was dissolved in distilled water and sterilized. CFSE-labeled CD8^+^T cells cultured in glucose-free medium (Gibco, Auckland, New Zealand, USA, No. 11879020) were incubated with 25 µM oleic acid and 12.5 µM palmitic acid or vehicle control for 72 hours.

To detect the effect of TAM on the proliferation of CD8^+^T cells, CFSE-labeled CD8^+^T cells were cultured alone or cocultured with the M2-polarized bone-marrow-derived macrophages (BMDMs) (at a 2:1 ratio) in 1640 medium supplemented with β-mercaptoethanol (0.05 mM), anti-CD3/CD28 dynabeads (Gibco, No. 11 452D), and colony-stimulating factor-1 (CSF-1) (20 ng/mL, Peprotech, No. 315–02) for 72 hours. The proliferation of T cells was measured by flow cytometry.

### Polarization of murine BMDMs

Bone marrow-derived monocytes were collected from 5 to 6 weeks old Balb/c mice and cultured in 1640 medium supplemented with 10% FBS and 20 ng/mL of CSF-1 for 6–7 days to obtain M0 BMDMs. Induction of M1 or M2 polarization was performed as previously described.^13^ CYH33 (1 µM) was incubated with BMDM for 1 hour before the addition of LPS (100 ng/mL, Sigma, No. L2880) or IL-4 (20 ng/mL, Peprotech, No. 214-14-20).

### RNA isolation and quantitative PCR

Total RNA was extracted using the RNeasy Mini Kit (Qiagen, No. 74106) according to the manufacture’s protocol. cDNA was synthesized with total RNA using a reverse transcription kit (Vazyme, Nanjing, China, No. 223–01). Quantitative PCR (qPCR) was performed with the cDNA and specific primers using SYBR Green Master Mix (Bio-Rad, California, USA, No. 172–5124). The primers used were listed in online supplemental table 2.

### RNA sequencing

RNA sequencing was performed by WuXi AppTec using the Illumina-HiSeq. Differentially expressed genes were identified with p value less than 0.01. Pathway enrichment analysis was carried out using GSEA, http://software.broadinstitute.org/gsea/index.jsp.

### Immunohistochemistry staining

Tumor tissues were fixed in 4% paraformaldehyde. Paraffin embedding, H&E staining and immunohistochemistry against CD45, CD4, CD8, CD11b, F4/80, CD206, cleaved-caspase 3, and Ki67 were conducted by Shanghai ZuoCheng Bio Company (Shanghai, China). Slides were observed under a Leica DM6 B microscope equipped with sCMOS camera (Leica, Wetzlar, Germany).

### Quantification of FFA in tumor tissue

Total lipids were extracted from tumor tissue as described.^34^ Briefly, tumor tissue was homogenized in methyl tert-butyl ether/methanol/water (5:1.5:1.45, v/v/v) and centrifuged at 15000×g for 10 min at 4℃. The organic phase was collected and vacuum dried. The lipid extracts were dissolved in methanol and subjected to the detection of FFA using LabAssay NEFA kit (Wako, Osaka, Japan, No. 294–63601) following the manufacture’s protocol.

### Statistical analysis

All statistical analyses were conducted using GraphPad Prism V.7 (La Jolla, California, USA) or R (V.3.5.1). Differences between two groups were calculated by unpaired two-tailed Student’s t-test. Differences among multiple groups were calculated by one-way or two-way analysis of variance. Differences were considered statistically significant when p value was less than 0.05. Data are presented as mean±SD unless otherwise noted. For each parameter of all data presented, *: p<0.05, **: p<0.01, ***: p<0.001, ****: p<0.0001.

## Results

### TME contributes to the activity of CYH33 to inhibit tumor growth in immune-competent mice

**Figure 1:**
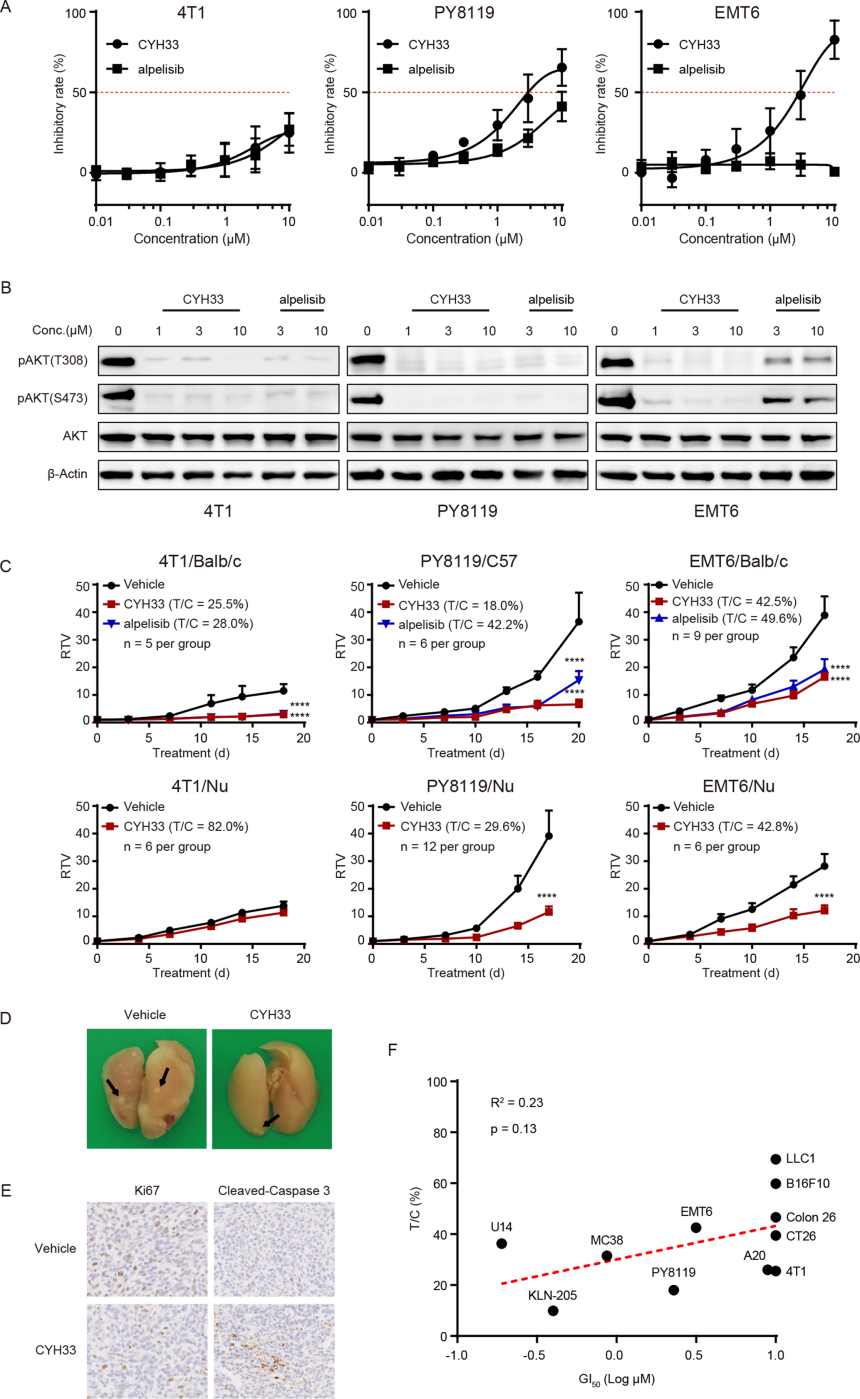
TME contributes to the activity of CYH33 to inhibit tumor growth in immune-competent mice. (A) 4T1, PY8119, and EMT6 cells were treated with CYH33 or alpelisib for 72 hours and cell proliferation was detected by sulforhodamine B (SRB) assay. The results were from three independent experiments. (B) 4T1, PY8119, and EMT6 cells were treated with CYH33 or alpelisib for 2 hours and cell lysates were subjected to Western blotting for the indicated proteins. The representative images were shown from two independent experiments. (C) Balb/c or C57BL/6 mice-bearing 4T1, EMT6 or PY8119 tumor allografts were orally administered with vehicle control, CYH33 (20 mg/kg, once a day), or alpelisib (50 mg/kg, once a day) for the indicated time. Nude mice bearing 4T1, EMT6, or PY8119 tumor allografts were administered with vehicle control or CYH33. Data are presented as mean±SEM. (D) Lungs were collected from Balb/c mice bearing 4T1 tumor at the end of the treatment and fixed with Bouin’s solution. The representative images were displayed and the nodules were indicated by the arrows. (E) The 4T1 tumor tissues were collected at the end of the treatment and subjected to immunohistochemistry to detect Ki67 and cleaved-caspase 3. The representative images were presented. (F) Pearson correlation analysis of the GI_50_s of CYH33 against cell proliferation in vitro and T/C values of CYH33 against corresponding allograft growth in vivo. RTV, relative tumor volume; T/C, treatment to control ratio; TME, tumor microenvironment. ****: p < 0.0001.

We previously reported that CYH33 overcame TAM-mediated resistance to radiation therapy, suggesting that CYH33 may execute its activity by reshaping the TME.^21^ To evaluate the antitumor activity of CYH33 in an immune-competent context, three lines of murine triple-negative breast cancer cells were used, namely 4T1, PY8119, and EMT6. As shown in figure 1A, CYH33 and alpelisib showed little activity against the proliferation of 4T1 cells with a GI_50_ over 10 µM. In comparison, CYH33 exhibited moderate antiproliferative activity against PY8119 and EMT6 cells with a GI_50_ of 2.29 µM or 3.16 µM, respectively. Consistent with previous studies,^20–22^ CYH33 is more active than alpelisib (figure 1A). The phosphorylation of AKT at Thr308 and Ser473 was almost completely blocked in the presence of CYH33 at concentrations of 1 µM or higher in three lines of cells (figure 1B), indicating that inhibition of PI3K/AKT pathway was not sufficient to suppress the proliferation of 4T1 cells.

To evaluate the efficacy of CYH33 in vivo, three lines of cells were inoculated to immune-competent or nude mice. CYH33 at 20 mg/kg markedly suppressed the growth of 4T1, PY8119 or EMT6 tumors in immune-competent mice with T/C values of 25.5%, 18.0% or 42.5%, respectively (figure 1C). Alpelisib at 50 mg/kg displayed similar or less potency compared with CYH33 (20 mg/kg) with T/C values of 28.0%, 42.2% or 49.6%, respectively. Notably, the therapeutic effect of CYH33 against 4T1 tumor were substantially impaired when cells were transplanted into nude mice with a T/C value of 82.0%, suggesting that the antitumor efficacy was associated with TME. CYH33 inhibited the growth of PY8119 and EMT6 allografts in nude mice as well with T/C values of 29.6% and 42.8%, respectively (figure 1C). Nonetheless, CYH33 appeared more potent in immune-competent mice than in nude mice, suggesting the partial contribution of the TME. In addition, the development of pulmonary metastasis in Balb/c mice bearing 4T1 allografts was reduced after CYH33 treatment (figure 1D, online supplemental figure S1A). A significant decrease in Ki67 and increase in cleaved-caspase 3 were observed on CYH33 treatment, indicating the inhibition of cell proliferation and induction of apoptosis (figure 1E, online supplemental figure S1B).

To determine the efficacy of CYH33 in tumors originating from other tissue types in immune-competent mice, a panel of murine tumor cells was screened in vitro and in vivo. As shown in figure 1F, online supplemental figures S1C and S1DCYH33 significantly attenuated the growth of allografts derived from lung cancer cells (KLN-205), colorectal cancers cells (MC38, CT26, Colon 26), B-cell lymphoma (A20), and cervical cancer cells (U14) with T/C values lower than 50%. Weak growth inhibition was observed in allografts from melanoma cells (B16F10) and lung cancer LLC1 cells. However, the activity of CYH33 in vitro represented as GI_50_ failed to be significantly correlated with that in vivo represented as the T/C value. This observation was consistent with that in 4T1 cells, indicating that CYH33 may execute its activity beyond direct action on tumor cells.

### CYH33 suppresses the proliferation of tumor cells in the immune-competent context

**Figure 2:**
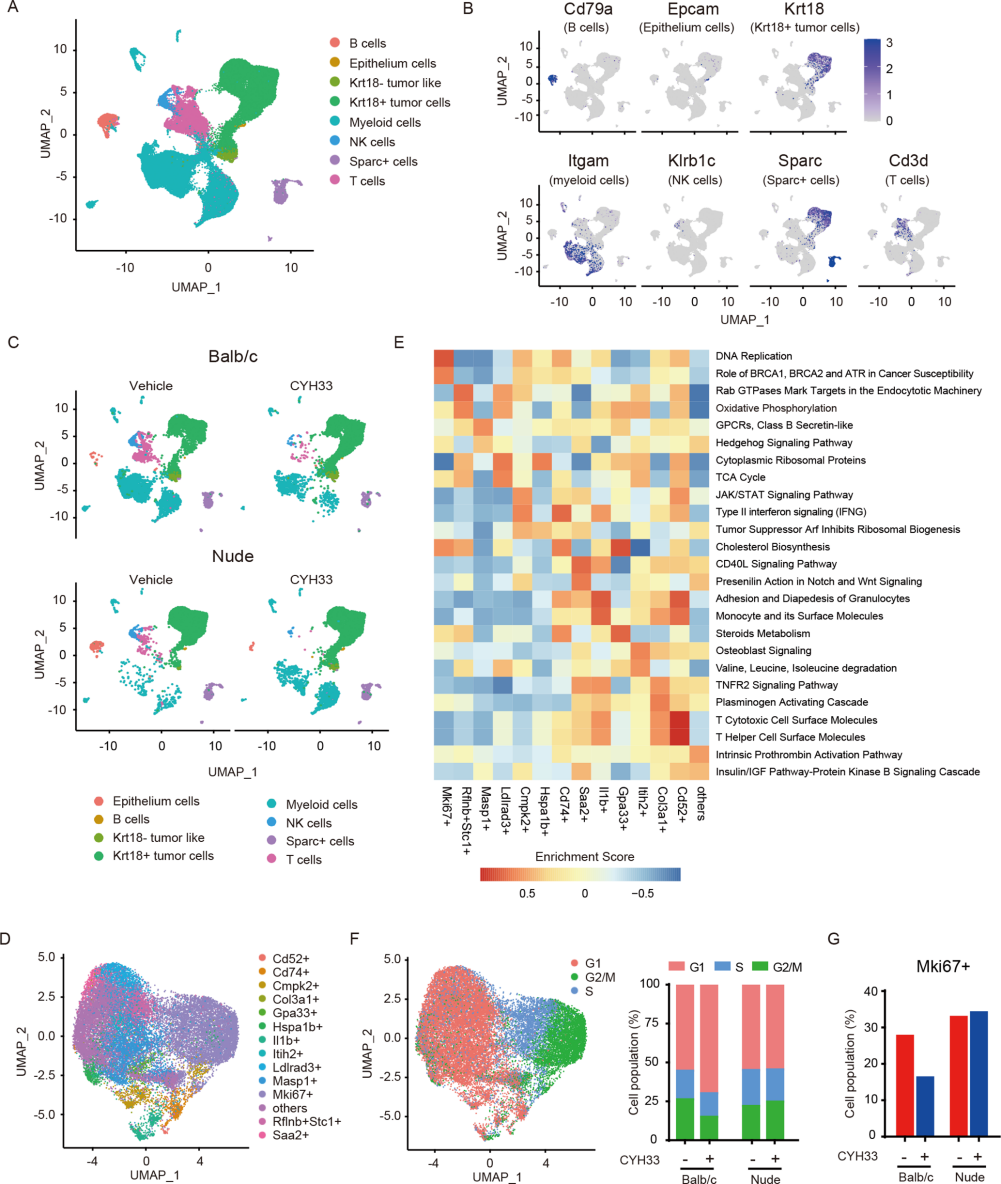
CYH33 suppresses the proliferation of tumor cells in the immune-competent context. (A) The four tumors including tumors from Balb/c mice treated with vehicle or CYH33, and tumors from nude mice treated with vehicle or CYH33 were harvested and subjected to scRNA-seq analyses, respectively. The data derived from four tumors were pooled to define the major cell clusters shown as UMAP plot. The distinct eight clusters labeled by inferred cell type were demarcated by colors based on distinctive biomarkers. (B) The normalized expression levels of established cell markers of eight clusters were shown by UMAP visualization. From left to right: Cd79a (B cells), Epcam (epithelium cells), Krt18 (Krt18^+^ tumor cells), Itgam (myeloid cells), Klrb1c (NK cells), Sparc (Sparc^+^ cells), and Cd3d (T cells). (C) The scRNA-seq data of each sample were analyzed to show the cellularity of the major cell clusters presented as UMAP plot. (D) UMAP plot of Krt18^+^ tumor cells. The Krt18^+^ cells were divided into 14 subpopulations by unsupervised clustering. (E) Gene set variation analysis was performed on the differentially expressed genes in different subcluster of Krt18^+^ tumor cells based on the curated pathway from gene set Knowledgebase database. The results were exported as a heatmap, and the two pathways with highest enrichment scores of each subpopulation were presented. (F) UMAP plot of Krt18^+^ tumor cells classified according to their cell cycle scores (left) and the percentage of cells assigned into different cell cycle phases (right). (G) The percentage of Mki67^+^ cluster in Krt18^+^ tumor cells. Krt18, keratin 18; scRNA-seq, single-cell RNA-seq; UMAP, Uniform Manifold Approximation and Projection.

**Figure 3:**
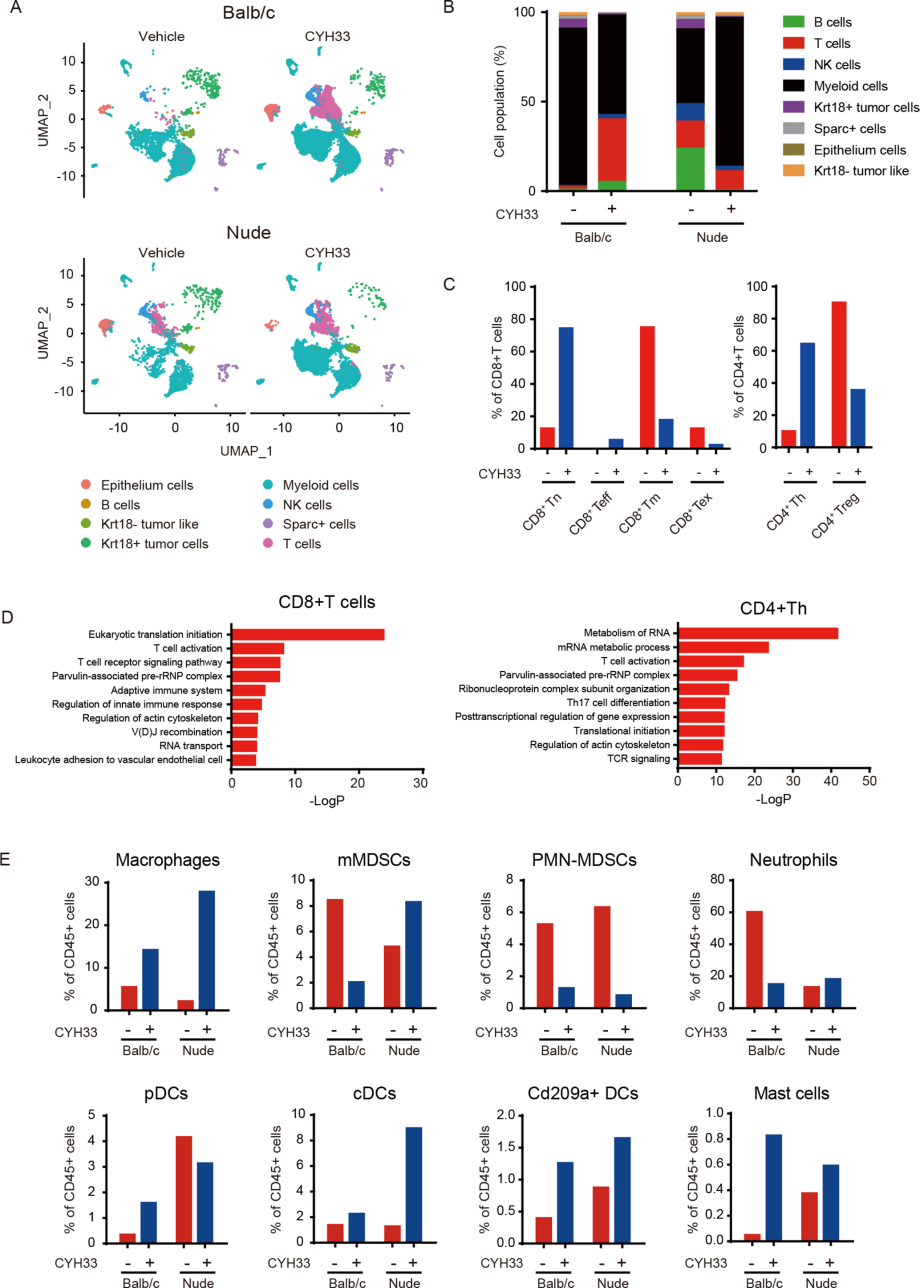
CYH33 reshapes the immune profile in the TME. (A) CD45^+^ immune cells were enriched respectively from the single-cell suspension of four tumor tissues as presented in figure 2A and subjected to scRNA-seq separately. UMAP plot of CD45^+^ immune cells from four tumor tissue samples was shown. (B) The percentage of each cluster in CD45^+^ immune cells was calculated. (C) The T cells from the enriched CD45^+^ cells in Balb/c mice were divided into six subclusters. The proportions of naïve CD8^+^T cells (CD8^+^ Tn), effector CD8^+^T cells (CD8^+^ Teff), memory CD8^+^T cells (CD8^+^ Tm), exhausted CD8^+^T cells (CD8^+^ Tex), helper CD4^+^T cells (CD4^+^ Th), and regulatory CD4^+^T cells (Treg) in CD3^+^CD8^+^T cells (left) or CD3^+^CD4^+^T cells (right) were presented. (D) The top-ranked enrichment pathways upregulated in CD3^+^CD8^+^T cells (left) or CD4^+^Th cells (right) from enriched CD45^+^ cells in Balb/c mice using genes with a fold change greater than three after CYH33 treatment. (E) The percentage of each subcluster of CD11b^+^ myeloid cells in CD45^+^ immune cells. cDCs, conventional dendritic cell; Krt18, keratin 18; MDSCs, myeloid-derived suppressor cells; pDC, plasmacytoid DCs; PMN, polymorphonuclear; scRNA-seq, single-cell RNA-seq; TME, tumor microenvironment; Treg, regulatory T cells; UMAP, Uniform Manifold Approximation and Projection.

We investigated the mechanisms underlying the differential activity of CYH33 in vitro and in vivo in the immune-competent context. Balb/c or nude mice bearing 4T1 allografts were treated with vehicle or CYH33 for 4 days (online supplemental figure S2A). The tumor tissues were digested to obtain a single-cell suspension. To generate a comprehensive transcriptional map of the cells, we performed scRNA-seq with the single cells. To define the major populations, the scRNA-seq data derived from the single cells of four tumors (34 474 cells) were pooled and unsupervised clustering was performed. Cells were classified into eight compositions visualized by Uniform Manifold Approximation and Projection (UMAP) (figure 2A, B, online supplemental figure S2B). Of these clusters, four populations of immune cells were identified. Tumor cells were identified with keratin 18 (Krt18). A cluster of cells was annotated as Krt18^−^ tumor-like cells, with an expression profile similar to that of Krt18^+^ cells but with low Krt18 expression. To determine the cluster distribution among different treatment groups, UMAP plot of each sample was shown (figure 2C) and the frequency of each cluster was presented (online supplemental figure S2C). The frequency of Krt18^+^ tumor cells did not decrease on CYH33 treatment in both Balb/c and nude mice. Therefore, the alterations in tumor cells need to be further investigated. Considering the low frequency of CD45^+^ immune cells in tumor tissue, the analyses of immune cells were then performed with the enriched CD45^+^ cells (figure 3).

To further characterize the feature of tumor cells, Krt18^+^ tumor cells were classified into 14 subclusters named by their uniquely expressed gene markers (figure 2D, online supplemental figure S2D). To reveal the characteristics of each subcluster, we performed Gene Set Variation Analysis on the differentially expressed genes in 14 subpopulations (figure 2E). The distinct markers and diverse enriched pathways of each cluster revealed high intratumoral heterogeneity (figure 2E). As rapid proliferation represents a hallmark of tumor cells, a subcluster was distinguished by the high expression of Mki67. Consistently, the DNA replication pathway was enriched in these Mki67^+^ cells (figure 2E). Cell cycle analysis was subsequently performed with the Krt18^+^ tumor cells. As shown in figure 2F, most of the Mki67^+^ cells coincided with the cell population at S or G_2_/M phase, confirming their proliferative state. The cell cycle distribution of the tumor cells indicates that the cell population in G_1_ phase increased in CYH33-treated Balb/c mice (figure 2F), suggesting that G_1_ phase arrest might be induced.^20 24^ No alterations were found in athymic mice (figure 2F). In parallel, although CYH33 did not downregulate the percentage of Krt18^+^ cells, the proportion of Mki67^+^ cluster in Krt18^+^ cells decreased in Balb/c mice but not in nude mice on CYH33 treatment, indicating that tumor cells are less proliferative in immune-competent mice (figure 2G). Taken together, the scRNA-seq analyses of tumor cells revealed the intratumoral heterogeneity of 4T1 allograft, and that treatment of CYH33 resulted in cell cycle arrest and a decrease in highly proliferative Mki67^+^ cells in immune-competent mice.

### CYH33 reshapes the immune profile in the TME

The differential in vitro and in vivo activity of CYH33 indicates the involvement of TME for its efficacy. Therefore, we separated CD45^+^ leucocytes from the tumor tissue and scRNA-seq was performed (online supplemental figure S2A). The CD45^+^ cells were classified into eight compositions using the same cell type annotations as presented in figure 2A (figure 3A). In immune-competent mice, the infiltration of T lymphocytes, B lymphocytes, and NK cells was significantly elevated on CYH33 treatment, which was not observed in nude mice (figure 3B, online supplemental figure S2E). In contrast, the proportion of myeloid cells markedly decreased in CYH33-treated Balb/c mice (figure 3B, online supplemental figure S2E). These data indicate that CYH33 modulates the profile of tumor-infiltrating leucocytes in the 4T1 tumor.

To further investigate the impact of CYH33 on T lymphocytes, CD3^+^T cells were clustered into six subpopulations by unsupervised clustering (online supplemental figure S3A–C). The proportion of each subcluster in CD3^+^CD8^+^ or CD3^+^CD4^+^ cells was calculated (figure 3C). The T cells from nude mice were not included in the analysis because of their anergy. Compared with the vehicle group, the proportion of naïve CD8^+^T, effector CD8^+^T, and helper CD4^+^T cells in the CYH33-treated group significantly increased, whereas the frequency of other subclusters including memory CD8^+^T cells, exhausted CD8^+^T cells, and protumoral Tregs was downregulated (figure 3C). To further determine the gene expression profile of T cells, we performed enrichment analyses using genes with a fold change cut-off of 3 after CYH33 treatment. As shown in figure 3D, the expression of genes involved in immune activation including the T-cell receptor signaling pathway and T-cell activation pathway enhanced in both CD8^+^T and helper CD4^+^T cells.

To study the effect of CYH33 on myeloid cells, we performed unsupervised clustering on the CD11b^+^ myeloid cells, which were divided into eight major cell clusters and the remaining small subpopulations in an ambiguous state were combined into one cluster designated ‘others’ (online supplemental figure S3D–F). The frequency of the eight major clusters in CD45^+^ cells is shown in figure 3E. The proportion of monocytic MDSCs, polymorphonuclear MDSCs, and neutrophils significantly decreased on CYH33 treatment in the immune-competent context. In addition, the infiltration of three clusters of dendritic cells (DCs) including conventional DCs, plasmacytoid DCs (pDCs), and CD209a^+^ DCs was enhanced in CYH33-treated Balb/c mice. A similar tendency was observed in nude mice except for the pDC cluster. Taken together, the scRNA-seq analyses of CD45^+^ cells demonstrated that CYH33 may exert its antitumor activity by modulating the TME indicated by the enhanced infiltration and activation of T cells, as well as the reshaped profile of myeloid cells.

### CYH33 induces a proinflammatory TME and immunological memory

**Figure 4:**
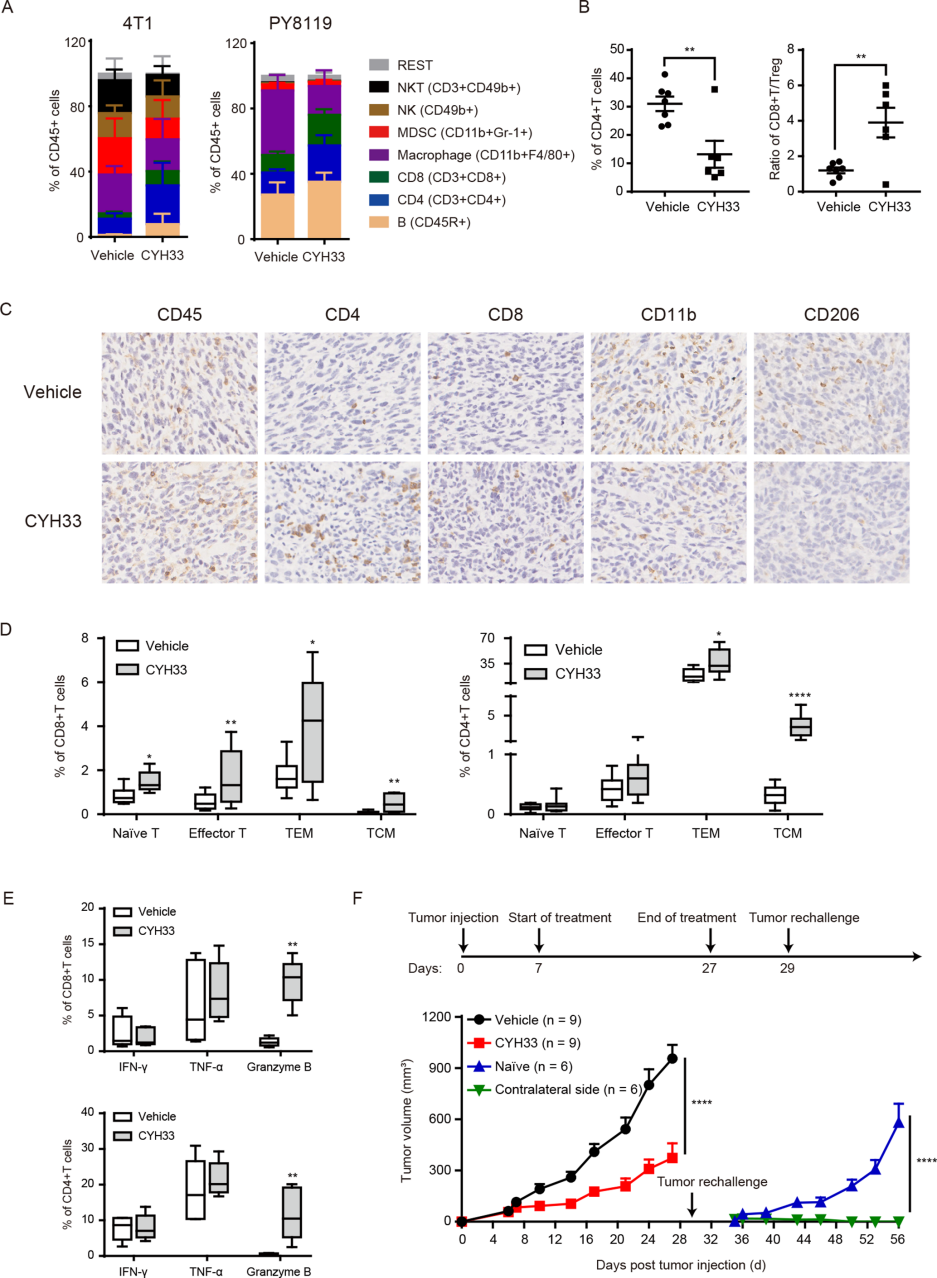
CYH33 induces a proinflammatory TME and immunological memory. (A) Immunophenotyping of 4T1 (left, n=9 in vehicle group, n=8 in CYH33-treated group) and PY8119 (right, n=5) tumors by flow cytometry after CYH33 treatment for 7 days. The percentage of indicated immune cells in CD45^+^ cells was presented. (B) The frequency of Tregs (left) and the CD8/Treg ratio (right) in 4T1 tumors. (C) Representative images of 4T1 tumor tissue sections that were collected after CYH33 treatment for about 3 weeks and immunostained with the indicated markers. (D) The percentage of naïve (CD45RA^+^CD62L^+^CD44^−^), effector (CD45RA^+^CD62^−^CD44^+^), effector memory (CD45RA^−^CD62^−^CD44^+^, TEM), and central memory (CD45RA^−^CD62L^+^CD44^+^, TCM) in the tumor-infiltrating CD8^+^T cells (left, n=9) or CD4^+^T cells (right, n=9). (E) The percentage of CD8^+^T cells (above) or CD4^+^T cells (bottom) positive of IFN-γ, TNF-α, or granzyme B (n=5). (F) The schedule of the tumor rechallenging experiment (above). The tumor volume was measured (bottom). Data are presented as mean±SEM. IFN-γ, interferon-γ; TCM, central memory T cell; TME, tumor microenvironment; TNF-α, tumor necrosis factor-α; Tregs, regulatory T cells. *: p < 0.05, **: p < 0.01, ****: p < 0.0001.

To further confirm the reprogramming of TME by CYH33, we examined the infiltration of the leucocyte population in the 4T1 allograft after CYH33 treatment for 7 days by multiparameter flow cytometry (online supplemental figure S4A). As shown in figure 4A, significant increases in the population of CD8^+^T cells, CD4^+^T cells, and B cells while reductions in MDSCs and macrophages were observed after CYH33 treatment. Because the TME appears to contribute partially to the activity of CYH33 in PY8119 tumors (figure 1C), we also performed immune profiling with PY8119 tumor allografts. The immune cells displayed different patterns in 4T1 and PY8119 tumors, which might be due to the different immunogenicity of the hosts or different recruitment of the immune cells by 4T1 or PY8119 cells. Nevertheless, CYH33 treatment displayed similar effect on the infiltration of immune cells in PY8119 tumors (figure 4A). The results further confirmed that CYH33 treatment induced remodeling of immune cells. Notably, the frequency of macrophage is inconsistent in flow cytometry and scRNA-seq analyses. The cells detected by scRNA-seq were derived from single mouse received vehicle or CYH33 treatment while the cells detected by flow cytometry were from eight mice in each group. Actually, the frequency of macrophage is relatively high in two of the eight mice in the CYH33-treated group in the flow cytometric analysis, but the frequency of macrophage decreased in most mice on CYH33 treatment. The appearing inconsistency may be caused by the individual variation in the infiltration of immune cells among mice. Considering the abundant sample size of mice analyzed in flow cytometric analysis, the results from flow cytometry may more accurately reflect the profile of immune cells. Tregs represent a distinct lineage of suppressive immune cells that could be targeted by PI3Kδ inhibitors.^17^ CYH33 treatment markedly decreased the infiltration of CD4^+^CD25^+^FoxP3^+^Tregs accompanied by an increased CD8/Treg ratio (figure 4B, online supplemental figure S4B). Similar results were observed in PY8119 tumors (online supplemental figure S4C). Consistently, immunohistochemistry demonstrated significant elevation in the staining of CD45, CD4, and CD8 (figure 4C, online supplemental figure S4D). We also observed a pronounced reduction in CD206, a marker for protumoral M2 macrophages (figure 4C, online supplemental figure S4D).

We further determined the effect of CYH33 on the profile of tumor-infiltrating T cells. As shown in figure 4D, the proportion of naïve, effector, effector memory (TEM), and central memory (TCM) CD8^+^T cells from the CYH33-treated group was markedly increased. Reduced expression of programmed cell death protein 1 (PD-1), which is the indicative marker of T-cell exhaustion, was observed in CD8^+^T cells (online supplemental figure S4E). These data further confirmed the results from scRNA-seq except for the memory subsets (figure 3C). In CD4^+^T cells, the frequency of TEMs and TCMs was significantly increased accompanied by the decreased expression of PD-1, whereas effector subsets were slightly increased and naïve subsets remained unchanged (figure 4D, online supplemental figure S4E). We further explored the expression of cytotoxic factors, including interferon γ (IFN-γ), tumor necrosis factor α (TNF-α) and granzyme B, in the tumor-infiltrating T cells. As shown in figure 4E, the intracellular level of granzyme B in CD8^+^T and CD4^+^T cells dramatically elevated after CYH33 treatment, demonstrating the enhanced cytotoxic activity of T cells. The levels of TNFα slightly increased and IFN-γ remained largely unchanged (figure 4E).

As infiltration of memory T cells increased after CYH33 treatment (figure 4D), we next explored whether CYH33 treatment motivated long-term memory. The mice-bearing primary 4T1 tumors were administered with vehicle or CYH33 for 20 days. CYH33-treated mice were then rechallenged with 4T1 cells on the contralateral side of flank. The same number of cells was inoculated to the naïve mice of the same age. In contrast to the allografts of naïve mice, the growth of the replanted 4T1 tumor was completely suppressed in mice previously treated with CYH33 (figure 4F). Collectively, these results suggest that a proinflammatory TME and immunological memory was induced in CYH33-treated 4T1 tumor.

### CD8^+^T cell is indispensable for CYH33 to inhibit 4T1 tumor

**Figure 5:**
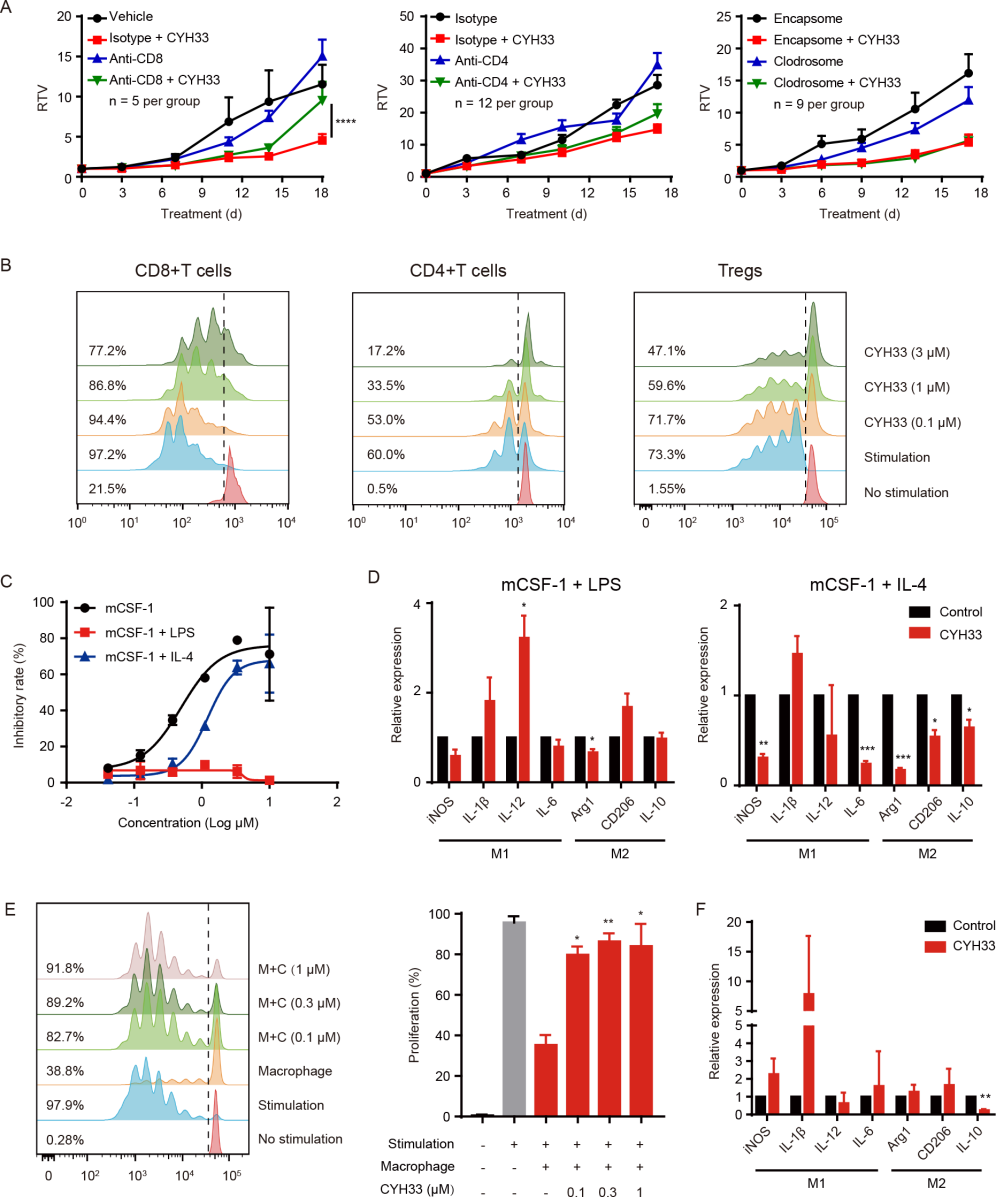
CYH33 inhibits 4T1 tumor progression via CD8^+^T cells and abrogates M2 macrophage-mediated suppression. (A) CD8^+^T cells, CD4^+^T cells, or macrophages were depleted in 4T1-bearing Balb/c mice by injection of anti-CD8 Abs, anti-CD4 Abs or clodrosome, respectively. The mice were administered with CYH33 for the indicated time. Data are presented as mean±SEM. (B) The CD8^+^T cells and CD4^+^T cells were isolated from spleens of naïve Balb/c mice and stimulated with anti-CD3 and anti-CD28. The Tregs were induced from CD4^+^CD25^-^T cells and stimulated with dynabeads coated with anti-CD3 and anti-CD28. The proliferation of CD8^+^T cells (left), CD4^+^T cells (middle) and Tregs (right) in the presence of CYH33 was determined by measuring the content of carboxyfluorescein succinimidyl amino ester (CFSE) with flow cytometry. Representative histograms depicting dividing cells were presented. The percentage of the dividing populations was indicated. The results were from two independent experiments. (C) The M0, M1 or M2-polarized BMDMs were induced by CSF-1, CSF-1 +LPS or CSF-1 +IL-4, respectively, for 24 hours and then treated with CYH33 for 72 hours. Cell viability was evaluated by the CCK-8 assay. (D) BMDMs were primed with LPS or IL-4 to induce polarization towards M1 (left) or M2 (right), respectively, in the presence of CYH33 at 1 µM. Total RNA were extracted 24 hours later and qPCR was performed to detect the mRNA level of indicated genes. (E) CD8^+^T cells labeled with CFSE were cocultured with M2-primed macrophages for 72 hours in the presence of CYH33. Representative histograms were shown (left) and percentage of dividing cells was calculated (right). (F) The mRNA level of indicated genes in macrophages cocultured with CD8^+^T cells was determined by qPCR. Data are presented as mean±SD from two independent experiments. IL-4, interleukin 4; qPCR, quantitative PCR; RTV, relative tumor volume; Treg, regulatory T cells. *: p < 0.05, **: p < 0.01, ***: p < 0.001, ****: p < 0.0001.

As CYH33 treatment resulted in enhanced infiltration and activation of T cell in tumor tissue, we investigated the roles of CD8^+^ and CD4^+^T cells in the activity of CYH33. CD8^+^T cells or CD4^+^T cells were depleted in tumor-bearing Balb/c mice by intraperitoneal (IP) injection of anti-CD8 or anti-CD4 antibodies, respectively (online supplemental figure S5A, B). The depletion of CD8^+^T cells significantly abrogated the inhibition of tumor growth by CYH33 (figure 5A). However, depletion of CD4^+^T cells marginally affected the efficacy (figure 5A), suggesting that inhibition of tumor growth by CYH33 may be predominantly dependent on CD8^+^T cells. As alteration in macrophage was also observed, we depleted macrophages by IP injection of clodrosome (online supplemental figure S5A, B). Depletion of macrophages mildly attenuated tumor growth, which was consistent with the role of TAMs in cancer development. The efficacy of CYH33 in clodrosome-treated mice (T/C value=47.3%) was slightly attenuated compared with the encapsome-treated group (T/C value=33.2%), indicating that macrophages may partially contribute to the activity of CYH33 (figure 5A).

To further investigate the modulation of immune cells by CYH33, we examined the effect of CYH33 on the proliferation of T cells. As shown in figure 5B and online supplemental figure S5C CYH33 marginally inhibited the proliferation of CD8^+^T cells. In contrast, CYH33 displayed significant antiproliferative activity in CD4^+^T cells (figure 5B, online supplemental figure S5C). Since CYH33 attenuated the infiltration of Tregs (figure 4B, online supplemental figure S4C), we further revealed that CYH33 significantly suppressed the cell division of Tregs (figure 5B, online supplemental figure S5C). Therefore, CD8^+^T cells are required for the efficacy of CYH33, which is not due to the direct effect of CYH33 on their proliferation.

### CYH33 abrogates M2 macrophage-mediated suppression on T-cell proliferation

We found that CYH33 decreased infiltration of total and M2-like macrophages in tumor tissue (figure 4A, C), suggesting that CYH33 may modulate the viability or polarization of macrophages. As shown in figure 5C, CYH33 preferentially suppressed the proliferation of M0 (IC_50_=0.704 µM) and M2-polarized (IC_50_=1.658 µM) BMDMs in a dose-dependent manner, whereas little effect on the proliferation of M1-polarized BMDMs was observed. We next detected the effect of CYH33 on the polarization of BMDMs toward M1 or M2, respectively (online supplemental figure S5D). As in figure 5D, CYH33-treated M1 macrophages displayed enhanced expression of IL-1β and IL-12. Conversely, the transcript levels of arginase 1, CD206, and IL-10 decreased in M2 macrophages. These data suggest that CYH33 may inhibit the proliferation of M2 macrophages and potentiate the macrophages to differentiate into a M1 phenotype.

M2-like TAMs abrogate antitumor immunity by impairing the expansion and activation of CD8^+^T cells.^35^ We next investigated whether CYH33 could alleviate the TAM-mediated suppression. M2 macrophages were co-cultured with CD8^+^T cells for 72 hours. M2 macrophages potently suppressed the proliferation of CD8^+^T cells, whereas the inhibition was significantly reversed by CYH33 in a dose-dependent manner (figure 5E). Consistently, CYH33 treatment enhanced the expression of iNOS and IL-1β, whereas the M2 marker IL-10 was markedly downregulated in co-cultured macrophages (figure 5F). These data demonstrate that CYH33 promotes the polarization of macrophages towards a M1-like phonotype, which may relieve CD8^+^T cells from M2 macrophage-mediated suppression.

### CYH33 treatment augments FA metabolism to boost antitumor immunity and synergizes with a FASN inhibitor

**Figure 6:**
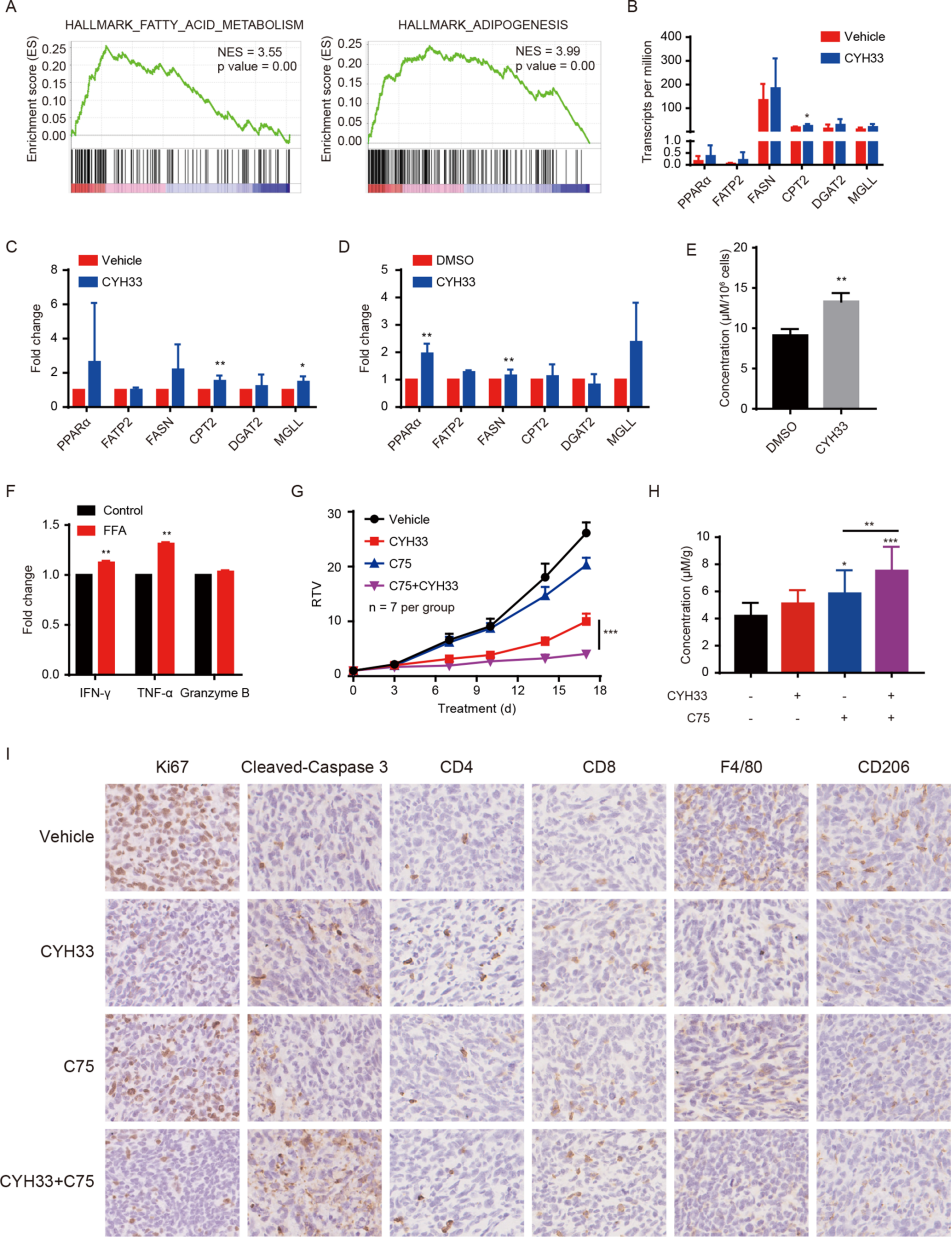
CYH33 treatment augments FA metabolism to boost antitumor immunity and synergizes with a FASN inhibitor. (A) The Balb/c mice bearing 4T1 tumors were treated with vehicle or CYH33 for 4 days and tumors were subjected to RNA-seq. Gene set enrichment analysis (GSEA) enrichment plots of differentially expressed genes in the gene set of FA metabolism and adipogenesis were presented. Red or blue represents positive and negative enrichment, respectively. (B) The transcripts per million of indicated genes involving in FA metabolism were presented (n=4). (C) The mRNA level of indicated genes from 4T1 allografts after CYH33 treatment for 4 days was measured by qPCR (n=5). (D) The mRNA level of indicated genes from 4T1 cells treated with 1 µM CYH33 for 24 hours was measured by qPCR. The results were from three independent experiments. (E) The level of FFA in the culture medium of 4T1 cells after treatment with 1 µM CYH33 for 72 hours from three independent experiments. (F) The expression of IFN-γ, TNF-α, and granzyme B in CD8^+^T cells cultured in the glucose-free 1640 medium with or without FFA supplementation were determined by flow cytometry. The results were presented as the fold change of median fluorescence intensity from two independent experiments. (G) Balb/c mice bearing 4T1 allografts were administered with vehicle control, CYH33 (20 mg/kg, once a day), C75 (10 mg/kg, once every 3 days), or combination of CYH33 and C75 for 17 days. The relative tumor volumes are presented as mean±SEM. (H) Tumor tissues were collected at the end of experiment and the level of FFA was measured from the lipid extraction (n=7). (I) Representative images of 4T1 tumor sections immunostained with Ki67, cleaved-caspase 3, CD4, CD8, F4/80 and CD206. FA, fatty acid; FFA, free fatty acid; FASN, FA synthase; IFN-γ, interferon-γ; qPCR, quantitative PCR; RTV, relative tumor volume; TNF-α, tumor necrosis factor-α. *: p < 0.05, **: p < 0.01, ***: p < 0.001.

To further elucidate the mechanism by which CYH33 modulates the TME, we profiled the transcriptome of 4T1 allografts with RNA-seq. GSEA of significantly differentially regulated genes demonstrated enhanced expression of genes associated with FA metabolism and adipogenesis (figure 6A). Furthermore, gene sets of ‘Hallmark_Bile_Acid_Metabolism’ and ‘Hallmark_Peroxisome’ were also unregulated on CYH33 treatment (online supplemental figure S6A), suggesting that CYH33 might reprogram the FA metabolism in tumor tissue.

To depict the gene expression profile, screening the genes involved in adipogenesis and FA metabolic processes indicated that FA uptake, FA oxidation, triglyceride synthesis, lipolysis, and adipogenesis were markedly elevated (online supplemental figure S6B). The transcripts per million values of six representative genes playing pivotal roles in FA metabolic processes are shown in figure 6B. The alteration in these genes was further confirmed by qPCR with the RNA extracted from 4T1 allografts (figure 6C). Similar results were obtained from 4T1 cells treated with CYH33 (figure 6D). CYH33 also significantly elevated the level of released FFA in cultured 4T1 cells (figure 6E). CD8^+^T cells may switch to FA catabolism to maintain their effector function in the glucose-deprived TME.^36^ To evaluate the impact of FFA on CD8^+^T cells in the nutrient-deficient microenvironment, CD8^+^T cells were cultured in glucose-free medium supplemented with FFA for 72 hours. The replenishment of FFA significantly enhanced the expression of IFN-γ and TNF-α, indicating that FFA may augment the cytotoxic activity of CD8^+^T cells under stressed condition (figure 6F). Nevertheless, the attenuated proliferation of CD8^+^T cells in the absence of glucose was not restored by the addition of FFA (online supplemental figure S6C).

FASN is the key metabolic enzyme of de novo lipogenesis that has been considered a therapeutic target in various cancer types.^37^ C75 is an inhibitor of FASN, and acts as a stimulator of carnitine palmitoyltransferase-1 to induce FA oxidation.^38^ To test whether C75 promotes the antitumor activity of CYH33, CYH33 and C75 alone or in combination were administered to 4T1 tumor-bearing Balb/c mice. Although C75 marginally inhibited tumor growth, the combination synergistically suppressed the tumor progression (figure 6G). However, CYH33 and C75 displayed no synergistic activity against 4T1 cells in vitro (online supplemental figure S6D), suggesting that the synergy in vivo requires the implication of TME. FFA in tumor tissue was significantly elevated after CYH33 treatment, which was further increased with the treatment of C75 alone or concurrent with CYH33 (figure 6H). The immunohistochemistry revealed that the combination therapy promoted the induction of apoptosis while inhibited the proliferation of tumor cells, which was accompanied by the enhanced infiltration of CD8^+^ T and CD4^+^T cells as well as the reduction of M2 macrophages (figure 6I). These data suggest that CYH33 may elicit antitumor immunity by augmenting FA metabolism and the combination of CYH33 with C75 synergistically inhibited 4T1 tumor growth with enhanced antitumor immunity.

## Discussion

The PI3K pathway plays pivotal roles in the multiple functions and biological processes of immune cells. Emerging evidence reveals the effect of targeting PI3K pathway on the tumor-infiltrating immune cells. Herein, we showed that activation of the host immune response by PI3Kα inhibitors contributed to their anti-cancer activity. The immunophenotyping of TME in the murine breast tumor revealed that CYH33 enhanced the infiltration and activity of T cells while abrogating suppressive immune cells. Mechanistically, CYH33 relieved the CD8^+^T cells from the immune suppression of M2-like macrophages and promoted FA metabolism in tumor tissue. The combination of CYH33 and FASN inhibitor exhibited synergetic activity accompanied by enhanced antitumor immunity.

For the first time, we comprehensively studied the effect of a PI3Kα-selective inhibitor on tumor cells as well as the immune microenvironment of solid tumors in an immune-competent context, and found that CYH33 exerted its activity by acting on both tumor cells and TME, as evidenced by the following observations. CYH33 was more potent in inhibiting tumor growth in immune-competent mice compared with athymic mice. By screening a panel of murine tumor cell lines, the activity of CYH33 in vitro failed to be significantly correlated with that in vivo, indicating that the in vivo efficacy was partially dependent on the TME. The in-depth profiling of cell population in tumor tissue by scRNA-seq and flow cytometry further revealed the alterations in the infiltration and function of immune cells, whereas the depletion of CD8^+^T cells significantly abrogated the efficacy of CYH33. Molecularly targeted drugs modulate the microenvironment and drive antitumor immunity.^39^ Selective inhibition of KRAS (G12C), which sits upstream of PI3Kα, induces a proinflammatory microenvironment with enhanced infiltration of T cells and cytokine production.^40^ CDK4/6 inhibitors promoted antitumor immunity by enhancing tumor antigen presentation, suppressing the Tregs, and activating CD8^+^T cells.^41 42^ These findings suggest that therapeutics originally targeting unlimited tumor cell proliferation may also act as immune modulators. A better understanding of their mechanisms of action on TME will facilitate their application in cancer therapy.

PI3K inhibitors regulate immune cells and influence the interaction between tumor and immune cells.^43^ Although CYH33 had minor effect on the proliferation of CD8^+^T cells directly, CYH33 significantly inhibited the proliferation of Tregs, consistent with the reduced tumor infiltration of Tregs (figures 4B and 5B). Selective targeting of PI3Kδ inhibits the proliferation of Tregs and attenuates their immunosuppressive function,^16 17^ in accordance with the dominant role of PI3Kδ in Tregs.^44^ Although CYH33 displayed the most potent activity against PI3Kα (IC_50_=5.9 nM) among class I PI3K isoforms, it strongly inhibited PI3Kδ kinase activity (IC_50_=78.7 nM) and PI3Kδ-medicated signaling at pharmacological concentrations,^23^ which may explain its inhibition of Treg proliferation. Interestingly, CYH33 also relieved macrophage-mediated suppression of the proliferation of CD8^+^T cells, which might be due to preferential polarization toward M1 instead of M2 in the presence of CYH33. These findings are in line with our previous report that CYH33 decreased the proportion of CD206-positive cells.^21^ Consistently, Thibault *et al*^45^ demonstrated that treatment with alpelisib in mice with pancreatic ductal adenocarcinoma decreased the infiltration of M2 macrophages. These results suggest that PI3Kα inhibitors may modulate the infiltration and polarization of TAM, yet the mechanism of action is obscure. Recent studies have demonstrated that PI3K inhibitors promote LPS/TLR4 signaling in macrophages,^46 47^ which is important for driving macrophages to a preferentially M1 phenotype.^48^ Alternatively, the metabolic state is closely linked to the differentiation of macrophages. Binding of FFA to TLR4 can activate the downstream activation of NF-κB and promote transcription of the proinflammatory genes.^49^ The elevated FFA levels by CYH33 might also affect the macrophages. The exact mechanism of action of CYH33 on the polarization of TAM needs to be further investigated.

The PI3K/AKT pathway plays important roles in a variety of metabolic processes by direct phosphorylation of metabolic enzymes or regulation of various transcription factors,^50 51^ which are important drivers of metabolic reprogramming in tumor cells.^52^ We found the enhanced expression of genes associated with FA metabolism and adipogenesis on CYH33 treatment. Although the reprogrammed metabolic state in 4T1 cells had little effect on cell proliferation, it appeared to modulate the TME. Augmented FA metabolic pathways lead to elevated FFA availability, which can serve as an important energy source for CD8^+^T cells in nutrient-deficient TME. Supplementation of FFA results in augmented expansion of CD8^+^T cells.^53^ Although the combination of C75 with CYH33 failed to inhibit the proliferation of 4T1 cells in vitro, this combination synergistically inhibited the growth of 4T1 allografts in vivo, which was accompanied by enhanced level of FFAs and improved infiltration of CD8^+^T cells in the tumor tissue. It is noteworthy that C75 treatment enhanced the FFA content while inhibited the FA synthesis. The level of FFA is determined by the balance between the anabolism and catabolism of cellular lipid. C75 treatment has been found to activate lipolysis and enhance the efflux of FFA.^54 55^ Increased FFA after C75 treatment found in this study may be ascribed to the activation of lipolysis. Moreover, C75 may also reprogram the metabolism of FA in immune cells, which also may contribute to the increased FFA in the tumor tissue. These results indicate that CYH33 may modulate the immune response by reprogramming metabolism and concurrent targeting of the metabolic pathway may improve the efficacy. However, the effect of PI3K inhibitors on global metabolome and subsequent immune modulation deserves further investigation. FASN has been considered an attractive therapeutic target in breast cancer and the FASN inhibitor TVB-2640 has shown preliminary activity in patients with advanced solid tumors including breast cancer in the phase I clinical study.^56^ The PI3Kα inhibitor alpelisib has been approved for the treatment of hormone receptor-positive breast cancer. The study provided a rationale to combine PI3Kα inhibitor with FASN inhibitor for the treatment of breast cancer. Meanwhile, the precise mechanism and safety profile of the combination needs to be systematically studied.

In conclusion, CYH33 elicited antitumor immune response against multiple tumors that originated from different tissue types in an immune-competent context via modulating the landscape of immune cells and reprograming the FA metabolism. Combination with C75 further promoted tumor immunogenicity, which demonstrated that the regulation of metabolism by PI3K inhibition could reshape the microenvironment. Our findings gain novel insights into how PI3K inhibitors exert their activity by modulating the tumor-immune interaction, and provide a rationale for the concurrent targeting of PI3K and FASN inhibitor in breast cancer treatment.

## Data Availability

Data are available on reasonable request. The datasets generated during the current study are available from the corresponding authors on reasonable request.
