# Supplementary material for: PI3Kα inhibitor CYH33 triggers antitumor immunity in murine breast cancer by activating CD8+T cells and promoting fatty acid metabolism
Source: J Immunother Cancer. 2021 Aug 9;9(8):e003093. doi: 10.1136/jitc-2021-003093 (PMC8354295; doi:10.1136/jitc-2021-003093)
Supplement: Supplementary data [file jitc-2021-003093supp001.pdf]

Supplemental Tables

Table S1. Fluorescently labeled antibodies for flow cytometry

**Name**	**Clone**	**Company**
APC-Cy7 Rat Anti-Mouse CD45	30-F11	BD Pharmingen
FITC Hamster Anti-Mouse CD3e	145-2C11	BD Pharmingen
BV605 Rat Anti-Mouse CD4	RM4-5	BD Pharmingen
PerCP-Cy™5.5 Rat Anti-Mouse CD8a	53-6.7	BD Pharmingen
BV650 Rat Anti-CD11b	M1/70	BD Pharmingen
APC Rat Anti-Mouse Ly-6G and Ly-6C	RB6-8C5	BD Pharmingen
PE-Cy7 Rat Anti-Mouse CD45R/B220	RA3-6B2	BD Pharmingen
BV421 Rat Anti-Mouse F4/80	T45-2342	BD Pharmingen
PE Hamster Anti-Mouse CD49b	HMα2	BD Pharmingen
BV650 Rat Anti-Mouse CD45RA	14.8	BD Pharmingen
PE-Cy7 Rat Anti-Mouse CD62L	MEL-14	BD Pharmingen
APC Rat Anti-Mouse CD44	IM7	BD Pharmingen
BV421 Hamster Anti-Mouse CD279 (PD-1)	J43	BD Pharmingen
APC Rat Anti-Mouse IFN-γ	XMG1.2	BD Pharmingen
Granzyme B Monoclonal Antibody (NGZB), PE-Cyanine7	NGZB	eBioscience

TNF alpha Monoclonal Antibody (MP6-XT22), APC	MP6-XT22	eBioscience
PE Rat Anti-Mouse Foxp3	R16-715	BD Pharmingen
BV421 Rat Anti-Mouse CD25	3C7	BD Horizon

Table S2 Primers used for qPCR

Gene name (Forward-F and Reverse-R)	Primer sequences
miNOS-F	CGTTGGATTTGGAGCAGAAGTG
miNOS-R	CATGCAAAATCTCTCCACTGCC
mARG1-F	GGAATCTGCATGGGCAACCTGTGT
mARG1-R	AGGGTCTACGTCTCGCAAGCCA
mCD206-F	TTGGACGGATAGATGGAGGG
mCD206-R	CCAGGCAGTTGAGGAGGTTC
mIL1β-F	GCAACTGTTCCTGAACTCAACT
mIL1β-R	ATCTTTTGGGGTCCGTCAACT
mIL12-F	GACATCACACGGGACCAAAC
mIL12-R	TACCAAGGCACAGGGTCATC
mIL6-F	CACAAGTCCGGAGAGGAGAC
mIL6-R	TTGCCATTGCACAACTCTTT
mIL10-F	AAGTGATGCCCCAGGCA
mIL10-R	TCTCACCCAGGGAATTCAAA
mCpt2-F	CAGCACAGCATCGTACCCA
mCpt2-R	TCCCAATGCCGTTCTCAAAAT
mFatp2-F	TCGTGGGACTGGTAGATTTTG
mFatp2-R	CGCGATGTGTTGAAAGAGTTT

mFasn-F	GGAGGTGGTGATAGCCGGTAT
mFasn-R	TGGGTAATCCATAGAGCCCAG
mPpara-F	AACATCGAGTGTCGAATATGTGG
mPpara-R	CCGAATAGTTCGCCGAAAGAA
mDgat2-F	GCGCTACTTCCGAGACTACTT
mDgat2-R	GGGCCTTATGCCAGGAAACT
mMgll-F	CGGACTTCCAAGTTTTTGTCAGA
mMgll-R	GCAGCCACTAGGATGGAGATG
mβ-Actin-F	TGTGATGGTGGGAATGGGTCAG
mβ-Actin-R	TTTGATGTCACGCACGATTTCC
mGapdh-F	CATGTTCCAGTATGACTCCACTC
mGapdh-R	GGCCTCACCCCATTTGATGT
