# Supplementary material for: PI3Kα inhibitor CYH33 triggers antitumor immunity in murine breast cancer by activating CD8+T cells and promoting fatty acid metabolism
Source: J Immunother Cancer. 2021 Aug 9;9(8):e003093. doi: 10.1136/jitc-2021-003093 (PMC8354295; doi:10.1136/jitc-2021-003093)
Supplement: Supplementary data [file jitc-2021-003093supp002.pdf]

Supplemental Figure S1

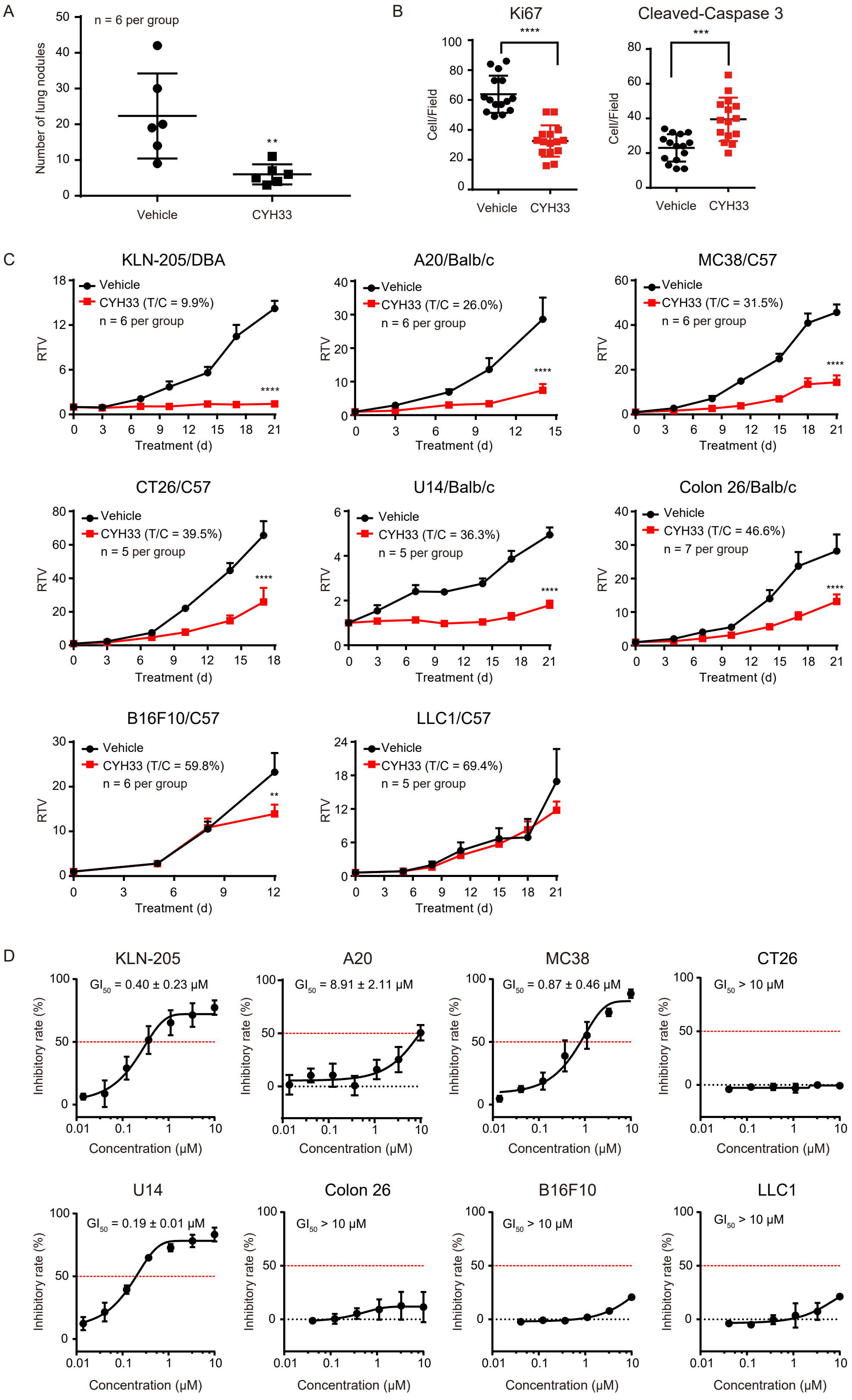

Supplemental Figure S1 (A) Quantification of the numbers of metastatic nodules in the lung tissues for Figure 1D. (B) Quantification of cells positive of Ki67 or cleaved-caspase 3 field of view within each mouse tumor for Figure 1E. (C) The anti-tumor activity of CYH33 in a panel of allografts derived from murine tumor cells. The tumor-bearing immune-competent mice received vehicle or CYH33 for the indicated time. Data are presented as mean ± SEM. (D) The indicated cells were treated with CYH33 for 72 h and cell proliferation was detected by SRB assay (KLN-205, MC38, CT26, U14, Colon 26, B16F10, and LLC1) or CCK-8 assay (A20). Data shown are mean ± SD from at least two independent experiments.

Supplemental Figure S2

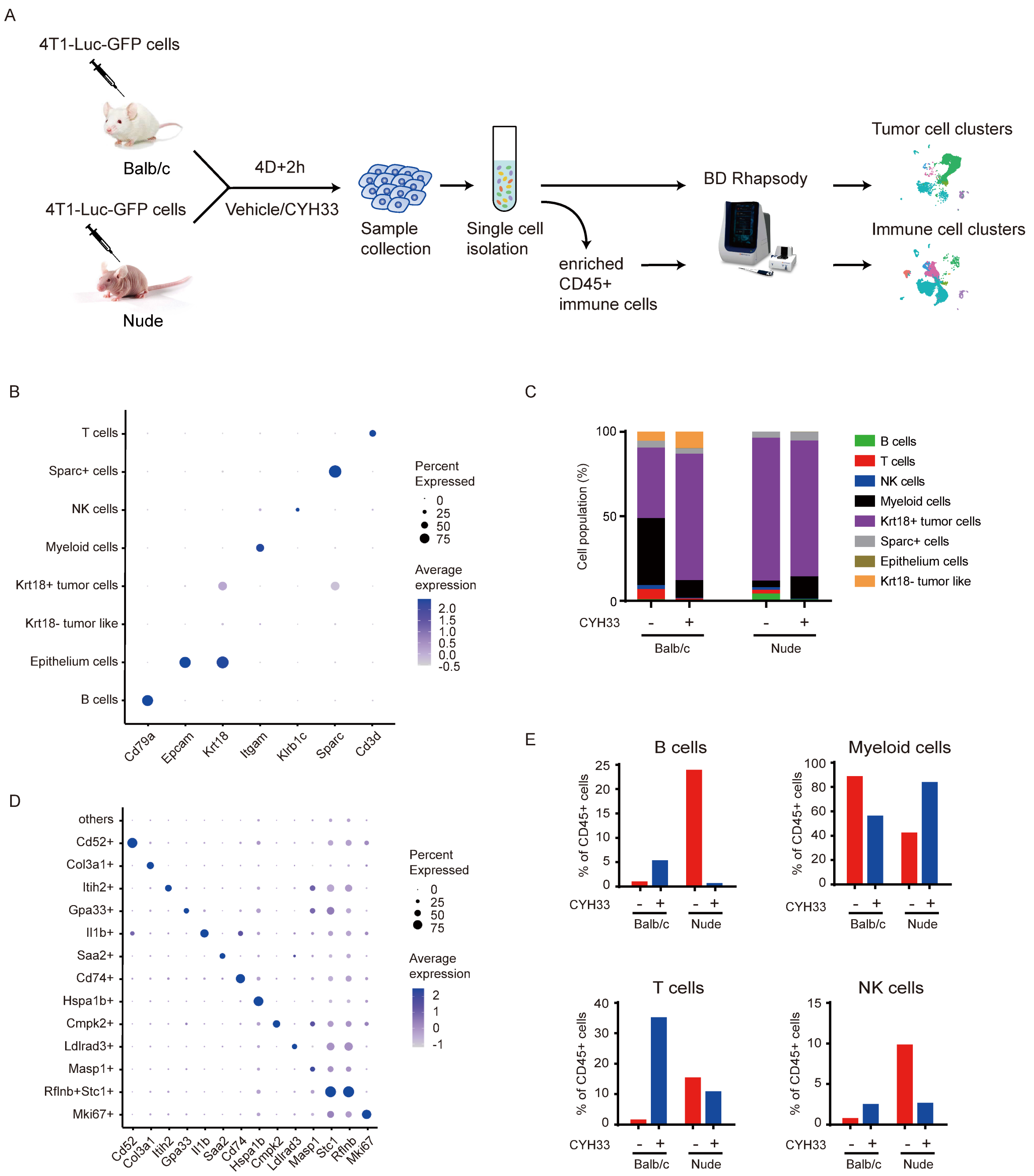

Supplemental Figure S2 (A) The schematic overview of sample preparation and scRNA-seq of tumor cells or enriched $CD45^+$ immune cells. (B) Dot plot for the expression of markers in the eight clusters in Figure 2A. Color represents normalized mean expression of markers in each cluster, and size indicates the proportion of cells expressing the marker. (C) The percentage of each cluster in the cells of 4T1 tumors from Balb/c or nude mice in Figure 2C. (D) Dot plot for expression of markers in distinct subsets of $Krt18^+$ tumor cells. (E) The proportion of B cells, myeloid cells, T cells, and NK cells in the $CD45^+$ immune cells.

Supplemental Figure S3

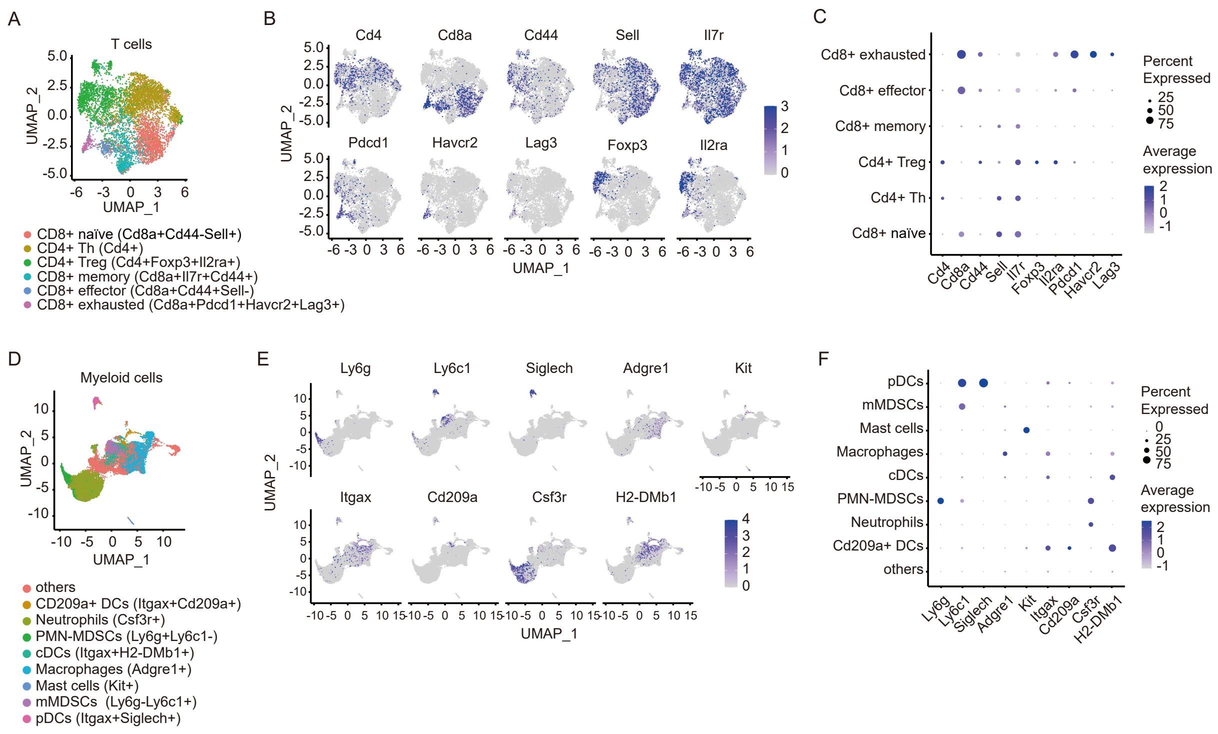

Supplemental Figure S3 (A) $CD3^{+}$T cells from $CD45^{+}$ cells enriched from 4T1 tumors in Balb/c mice were divided into distinct six subclusters as identified by unsupervised clustering. The UMAP plot was shown. (B) Expression level of established cell markers of subsets in $CD3^{+}$T cells was shown by UMAP plot. (C) Dot plot for expression of markers in distinct subsets of $CD3^{+}$T cells. (D) The myeloid cells from $CD45^{+}$ cells enriched from 4T1 tumors in Balb/c mice or nude mice were clustered and presented as UMAP plot. (E) UMAP plot of expression level of established cell markers of different subsets in $CD11b^{+}$ myeloid cells. (F) Dot plot for expression of markers in distinct subsets of myeloid cells.

Supplemental Figure S4

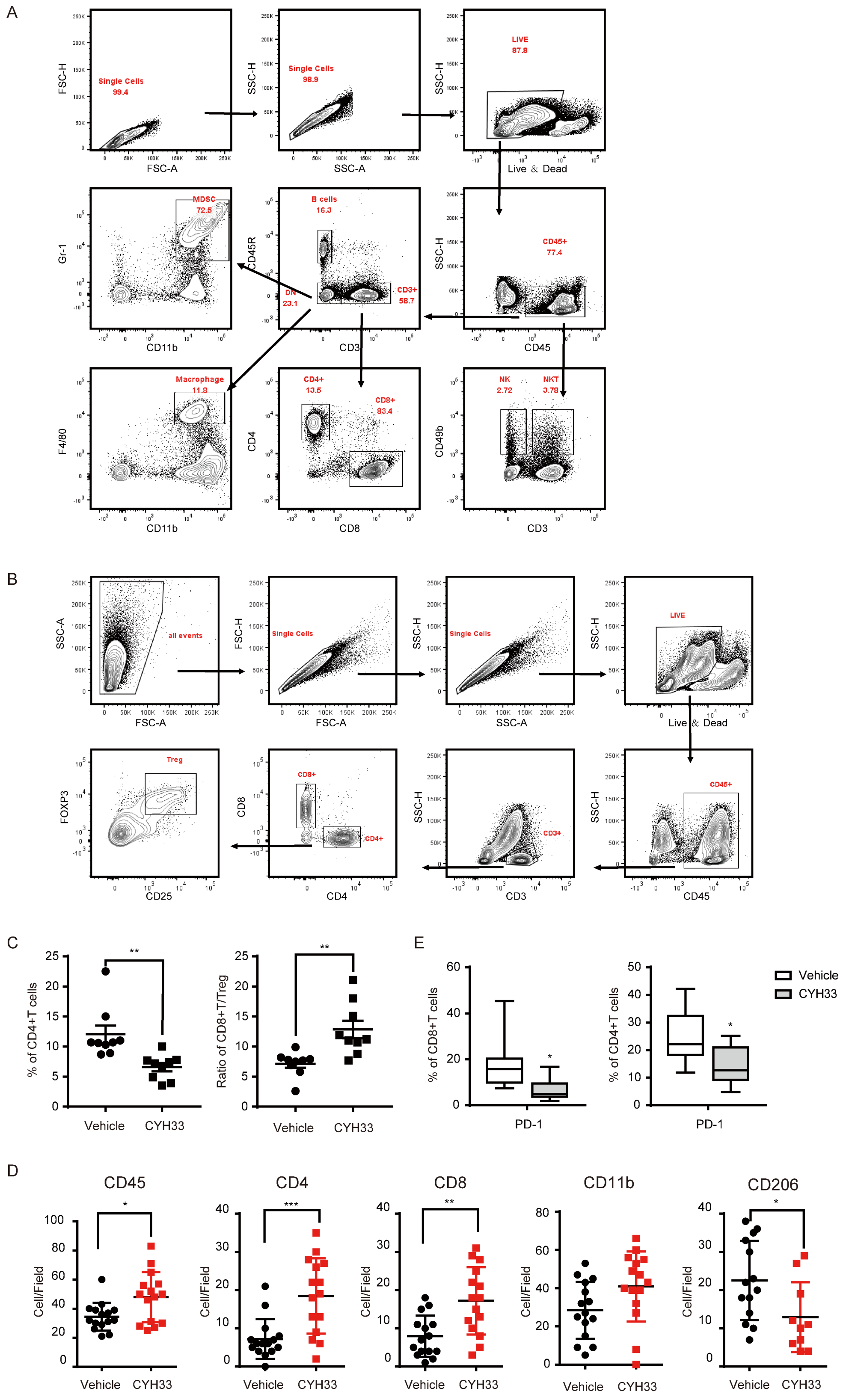

Supplemental Figure S4 (A) FACS gating strategy for intra-tumoral immune cells in 4T1 or PY8119 tumor allografts for Figure 4A. (B) FACS gating strategy for tumor-infiltrating Tregs for Figure 4B. (C) The frequency of Tregs measured by flow cytometry (left) and the CD8/Treg ratio (right) in PY8119 tumors. (D) Quantification of cells positive of CD45, CD4, CD8, CD11b or CD206 field of view within each mouse tumor for Figure 4C. (E) The proportion of $CD8^{+}$T cells (left) and $CD4^{+}$T cells (right) expressing PD-1 in 4T1 tumor allografts (n = 9).

Supplemental Figure S5

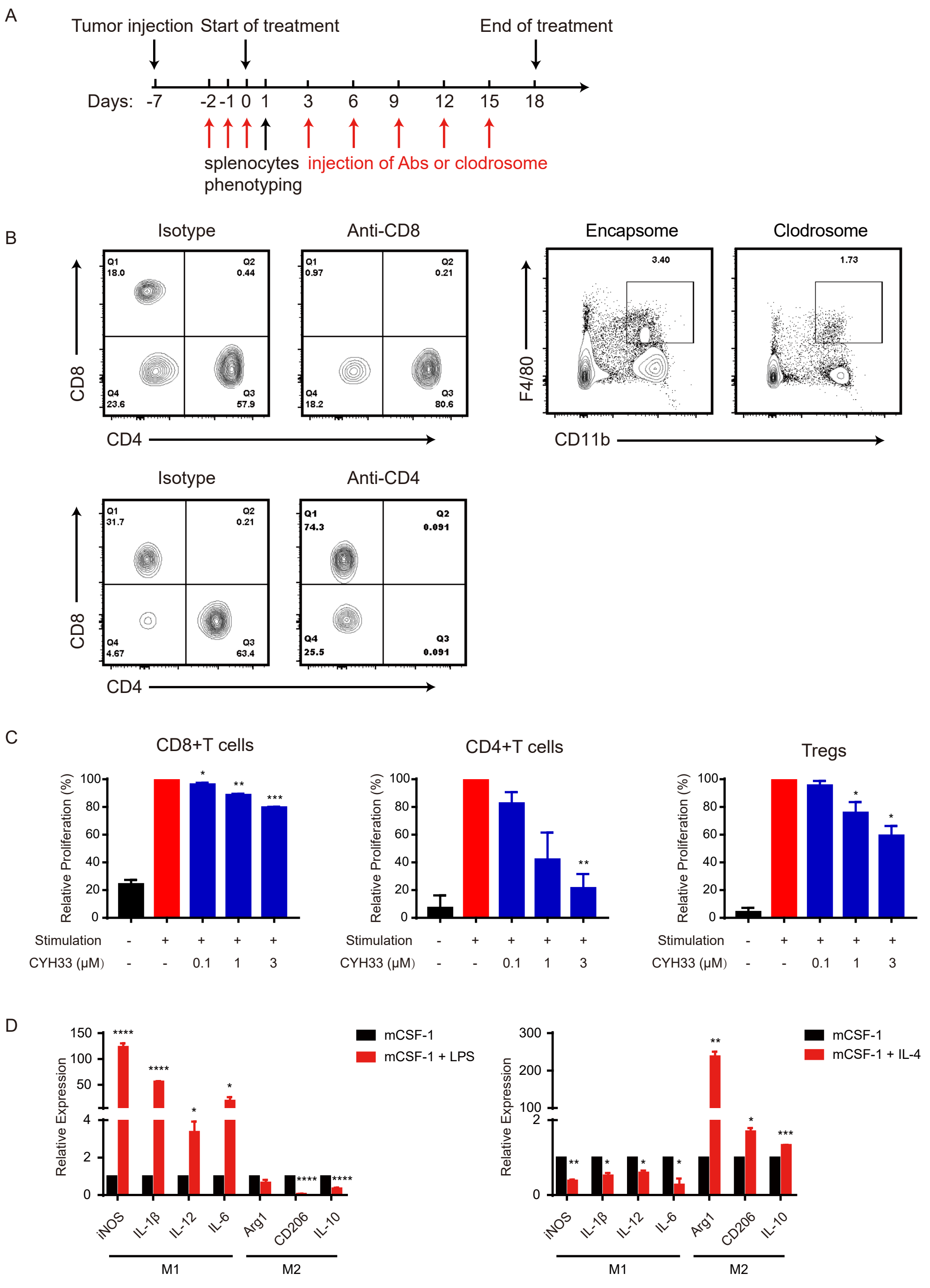

Supplemental Figure S5 (A) The schedule of the depletion of CD8$^+$T cells, CD4$^+$T cells, and macrophages. Anti-CD4 Abs, anti-CD8 Abs, or clodrosome were injected on days -2, -1, 0, 3, 6, 9, 12, and 15 as indicated by red arrows. The spleens were harvested at day 0 as indicated by black arrows. The splenocytes were subjected to flow cytometry to confirm the depletion of the corresponding immune cells. (B) The flow cytometric analysis of CD8$^+$T cells (upper left), CD4$^+$T cells (bottom), or macrophages (upper right) in the splenocytes isolated from tumor-bearing mice injected with anti-CD8 Abs, anti-CD4 Abs, or clodrosome, respectively. (C) Quantification of the proliferating CD8$^+$T cells (left), CD4$^+$T cells (middle), and Tregs (right) after CYH33 treatment. The results were from two independent experiments. (D) BMDMs were primed with LPS (left) or IL-4 (right). The mRNA levels of markers representing M1 or M2 macrophages were determined by qPCR. Data are presented as mean ± SEM from two or three independent experiments.

Supplemental Figure S6

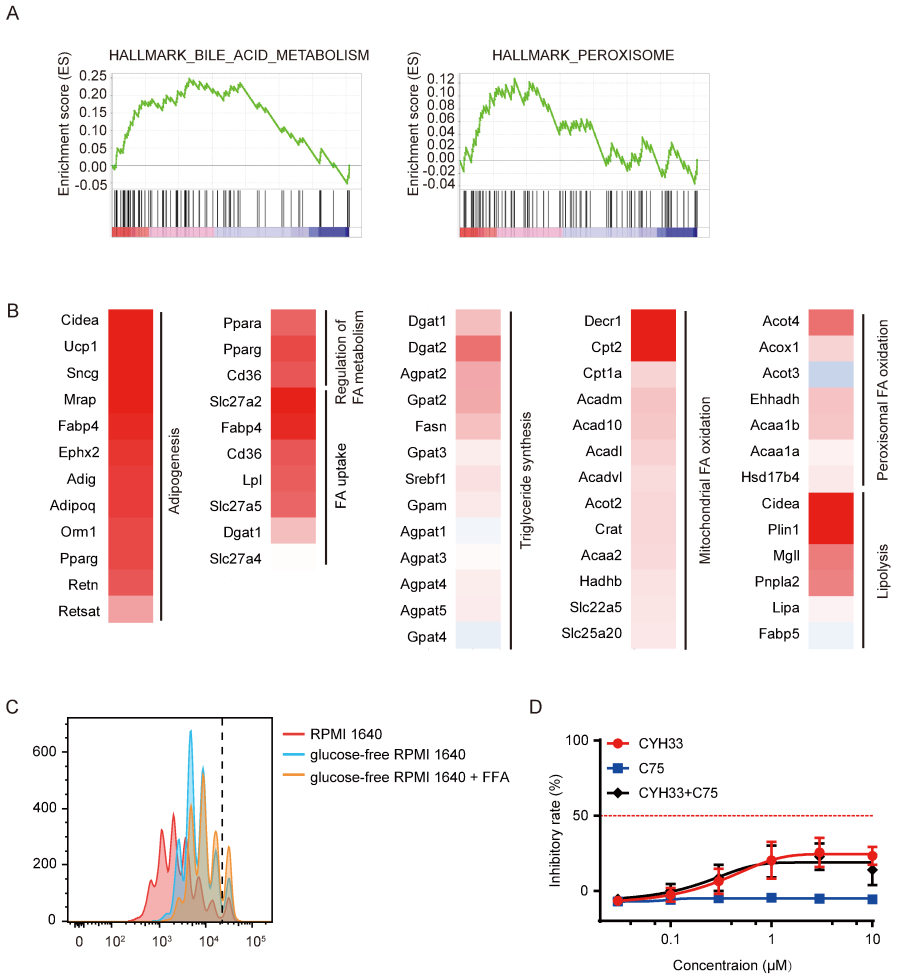

Supplemental Figure S6 (A) The Balb/c mice bearing 4T1 tumors were treated with vehicle or CYH33 for four days and tumors were subjected to RNA-seq. Gene set enrichment analysis was performed using the fold change of each gene in a comparison of CYH33 versus vehicle group. Red or blue represents positive and negative enrichment, respectively. The analysis showed the enrichment of differentially expressed genes in the gene set of bile acid metabolism and peroxisome. (B) The fold change of genes implicated in the adipogenesis, FA uptake, FA oxidation, triglyceride synthesis, and lipolysis were depicted as a heatmap. (C) $CD8^+$T cells labelled with CFSE were cultured in the regular, glucose-free, or glucose-free supplemented with FFA medium for 72 h. Representative histograms depicting dividing cells from two independent experiments were presented. (D) 4T1 cells were treated with CYH33, C75, or combination of CYH33 and C75 for 72 h. Cell proliferation was detected by SRB assay. The results were from two independent experiments.
